# Co-expression of *Cassia tora* 1-deoxy-D-xylulose-5-phosphate synthase and 1-deoxy-D-xylulose-5-phosphate reductoisomerase enhances tolerance of transgenic *Nicotiana benthamiana* to lead (Pb) stress

**DOI:** 10.3389/fpls.2025.1657368

**Published:** 2025-11-04

**Authors:** Xue Huang, Chunyao Tian, Zichun Ma, Jieru Chen, Hongting Liu, Wei Zhang, Zhongda Li, Pingping Lu, Leyao Wang, Hai Liao, Jiayu Zhou

**Affiliations:** 1 School of Life Science and Engineering, Southwest Jiaotong University, Chengdu, Sichuan, China; 2 Bureau of Longnan Business Environment Construction, Longnan, Gansu, China

**Keywords:** ABA, Cassia tora, Pb stress, MEP pathway, Nicotiana benthamiana

## Abstract

Lead (Pb) stress causes impairment of plant growth and loss in crops. Exogenous addition of abscisic acid (ABA) could alleviate Pb damage, however, the roles of genes involved in the biosynthesis of ABA in Pb tolerance were still unclear. In this study, we found that the transcription of *Cassia tora 1-deoxy-D-xylulose-5-phosphate synthase 1* (*CtDXS1*) and *1-deoxy-D-xylulose-5-phosphate reductoisomerase 1* (*CtDXR1*) genes was upregulated by lead acetate (Pb). Subsequently, we evaluated the anti-Pb effects of *Nicotiana benthamiana* coexpressing *CtDXS1* and *CtDXR1* genes. The transgenic lines conferred improved performance under Pb stress, such as more endogenous ABA content and higher antioxidant enzyme activities, but lower levels of malonaldehyde (MDA) and H_2_O_2_ contents as well as Pb uptake than in the wild-type plants. Additionally, the role of ABA in Pb tolerance was verified. The transcript of heavy metal-tolerant genes, such as ABC transporters and ATPase, were enhanced in the transgenic plants, with auxin transporter protein 1 (AUX1) and calcium-binding protein CP1 (CP1) being potential key nodes in Pb-tolerant signaling network. In addition, Pb-tolerant microbes such as genera *Methylophilus*, *Massilia* and *Bradyrhizobium* were enriched in the rhizosphere microbial community of transgenic plants. To our knowledge, this first report demonstrating 2-*C*-methyl-D-erythritol-4-phosphate (MEP) pathway-mediated accumulation of ABA confers Pb tolerance.

## 1 Introduction

In contemporary times, global heavy metal contamination has been escalating due to the increased production of industrial, agricultural, anthropogenic, and natural wastes (Kumar and Prasad, 2018). Lead (Pb), identified as the second most toxic heavy metal after arsenic (As), serves no essential biological function in living systems (Usman et al., 2020; Zulfiqar et al., 2019). The detrimental impacts of Pb on plants are primarily driven by the excessive production of oxidative stress (ROS), which in turn causes oxidative damage to biological macromolecules, cellular components, and tissue ultrastructure (Tian et al., 2023). Consequently, Pb, in conjunction with ROS, significantly inhibits photosynthetic processes (Zhang et al., 2024a), disrupts DNA integrity (Kaur et al., 2021), impairs nutrient uptake (Chandwani et al., 2023), and undermines overall plant performance (Kumar and Prasad, 2018), thereby exerting adverse effects on growth and crop productivity (Ashraf et al., 2020). Moreover, the uptake and accumulation of Pb in crop plants lead to chronic dietary exposure in humans, which is associated with a spectrum of health risks (Clemens, 2019). Toxic deposits of Pb in the human body also trigger oxidative stress, resulting in severe substantial neuropsychological and functional disorders (Babić et al., 2022; Naranjo et al., 2020). The growing urgency of concerns regarding food and ecosystem safety has thus spurred intensive efforts to enhance plant tolerance to Pb pollution.

In response to diverse abiotic stresses, including Pb stress, plants have evolved sophisticated adaptive mechanisms involving physiological, biochemical, and molecular alterations. Among these, several phytohormone-mediated pathways directly contribute to plant protection against abiotic stress and maintain cellular homeostasis. Abscisic acid (ABA) is a pivotal hormone in response to a multitude of abiotic and biotic stresses (Xu et al., 2022). Specifically, the application of exogenous ABA mitigates Pb toxicity by facilitating the translocation of Pb from root to shoot in *Populus* × *canescens* (Shi et al., 2019). In cucumber and wheat, exogenous ABA enhances cold tolerance and Ca(NO_3_)_2_ tolerance by boosting the activities of antioxidant enzymes (Shu et al., 2016; Yu et al., 2020). Additionally, ABA induces the expression of genes harboring ABA-responsive *cis*-elements (ABREs) in the promoters of various stress-related genes, thereby enhancing tolerance to diverse abiotic stresses (Vashisth et al., 2024). Owing to its role in plant defense, ABA metabolism has garnered increasing research attention. As a 15-carbon isoprenoid, ABA is primarily derived from the cleavage of C40 carotenoids mainly through the 2-*C*-methyl-D-erythritol-4-phosphate (MEP) pathway in the plastids (Milborrow, 2001). Initiating from pyruvate and glyceraldehyde 3-phosphate, the MEP pathway is ubiquitously distributed across organisms ranging (e.g., *E. coli* and cyanobacteria) to higher plants (Estévez et al., 2001). Prior studies have illustrated that genes in the MEP pathway play crucial roles in plant responses to various stresses, including drought, salinity, and fungal infections, by regulating the accumulation of downstream isoprenoid products, such as carotenoids and ABA (Tian et al., 2023; Wei et al., 2019; Xu et al., 2019). However, to date, the specific roles of these genes in conferring tolerance to heavy metal stress remain unelucidated.

*Cassia tora* (*Senna tora*), an annual medicinal plant, has been utilized for over 1800 years in China to treat ailments such as headaches, dizziness, and hypertension (Chen et al., 2024). Beyond its pharmaceutical applications, *C. tora* has demonstrated inherent metal tolerance, likely due to its capacity to alleviate oxidative stress (Li et al., 2009; Wang and Yang, 2005). In our previous research, we identified two genes, *C. tora* DXS1 (*CtDXS1*, accession number of KAF7813697.1) and DXR1 (*CtDXR1*, accession number of KAF7806089.1), which encode the first and second enzymes in the MEP pathway, respectively (**Supplementary Figure S1**). These genes were found to participate in ABA biosynthesis and confer drought tolerance (Tian et al., 2023). Furthermore, ABA has been shown to correlate with the elevated transcriptional levels and activities of antioxidant enzymes such as peroxidase (POD) and superoxide dismutase (SOD) (Shu et al., 2016; Yu et al., 2020). Based on these findings, we hypothesized that the *CtDXS1* and *CtDXR1* genes might also contribute to Pb tolerance. Therefore, the aim of this study was to investigate the performance of plants co-overexpressing these genes under Pb stress, and accordingly, a potential molecular mechanism underlying the Pb tolerance phenotype was put forward.

## 2 Materials and methods

### 2.1 Expression patterns of *CtDXS1* and *CtDXR1* under Pb stress

To investigate the expression responses of *CtDXS1* and *CtDXR1* genes under Pb stress, sterile seedlings of *C. tora* (7-day-old post-germination) were cultured in Murashige and Skoog (MS) solid medium at 25°C under a 16/8-h light/dark photoperiod. The seedlings were subsequently subjected to MS medium supplemented with 0.5 mmol/L Pb acetate for durations of 0, 1, 2, 3, 6, 12, 24, and 48 hours. Gene-specific primer pairs for *CtDXS1* (CtDXS1-qF/CtDXS1-qR in **Supplementary Table S1**), *CtDXR1* (CtDXR1-qF/CtDXR1-qR in **Supplementary Table S1**), and the reference gene *Elongation factor 1a* (EF1α2-qF and EF1α2-qR in **Supplementary Table S1**) were employed as previously reported (Tian et al., 2023). Real-time PCR analysis was performed using a LightCycler 96 system (Roche Diagnostics, Germany) with SYBR Premix Ex Taq II (Takara, Japan). Relative gene expression levels were calculated via the ΔΔCT method (Liao et al., 2023).

### 2.2 Construction of *CtDXS1_CtDXR1* overexpressing plasmid

To generate transgenic plants exhibiting co-expression of these genes, a modified pCambia1301 binary vector containing two separate expression cassettes (expression cassette A and B) was utilized. In the first step, the full-length open reading frames (ORFs) of the *CtDXS1* gene were amplified by PCR using the CtDXR1-B-F/CtDXR1-B-R primer pair containing *Bgl*II/*BstE*II site, respectively (**Supplementary Table S1**). The amplified PCR product was then cloned into expression cassette A of the vector, which had been previously digested with *Bgl*II and *BstE*II and subsequently gel purified. This vector was named pCambia1301: *CtDXR1*. Subsequently, the full-length ORF of the *CtDXS1* gene was PCR amplified using the forward primer CtDXS1-K-F containing *Kpn*I site and reverse primer CtDXS1-K-R containing *Kpn*I site from **Supplementary Table S1**. The pCambia1301: *CtDXR1* vector was linearized with *Kpn*I and then ligated with the *CtDXS1* fragment in expression cassette B using homology recombination via the ClonExpress-II One Step Cloning Kit (Vazyme, China), resulting in the creation of the dual-transgene expression vector pCambia1301: *CtDXR1_CtDXS1* plasmid. The accomplishment of recombinant plasmid was confirmed by sequencing and PCR amplification. The schematic diagram of vector construction was listed in **Supplementary Figure S2**.

### 2.3 Transformation and selection of *Nicotiana benthamiana* transformants

To achieve co-expression of the *CtDXS1* and *CtDXR1* genes, the recombinant vector was firstly introduced into *E. coli* DH5α, followed by transformation into *N. benthamiana* via Agrobacterium-mediated method. Putative transgenic plantlets were selected on selection medium supplemented with hygromycin, under controlled growth chamber conditions, in accordance with the antibiotic resistance marker gene present in the plasmid (Tian et al., 2023). Genomic DNA was extracted from surviving lines and used as template for PCR verification of transgenic integration. The primer pairs, CtDXS1-K-F/CtDXS1-R and CtDXR1-F/CtDXR1-R, were listed in **Supplementary Table S1**. The T2 generation transgenic plants demonstrated a green phenotype similar to wild-type controls and were subjected to analysis of mRNA levels, isoprenoid content, and phenotype traits.

### 2.4 Determination of carotenoid, ABA, chlorophyll, and GA_3_ contents

The contents of carotenoids, ABA, GA_3_, and chlorophyll in leaves of wild-type and transgenic plants were quantified using established methods (Tian et al., 2023). For chlorophyll and carotenoid analysis, 0.1 g of leaf tissues were homogenized in 1 ml of 95% ethanol using a high-speed cryogenic grinder. The homogenate was centrifugated, and the supernatant was collected and diluted to a final volume of 1 ml with 95% ethanol. Absorbance was measured at 470 nm, 649 nm, and 665 nm using a Shimadzu UV-2200 spectrophotometer. For ABA and GA_3_ extraction, fresh leaf tissues were macerated in 80% ethanol and stirred continuously for 16 hours at 4°C. The supernatant was concentrated via centrifugation and re-extracted with ethyl acetate, and evaporated under vacuum to a volume of 1 mL of 80% methanol. After filtration through a 0.22 µm, 13 mm membrane filter (Millipore), ABA and GA_3_ were analyzed simultaneously by HPLC on a Hypersil C18 column (4.6 mm × 150 mm) based on controlled method. Each experiment was conducted with three biological replicates.

### 2.5 Assays of antioxidant enzyme activities

Briefly, 500 mg of leaf tissue was ground to a fine powder in liquid nitrogen and extracted in 50 mmol/L sodium phosphate buffer (pH 7.8) containing 1% (w/v) polyvinylpolypyrrolidone (PVPP) and 0.1 mol/L EDTA-Na_2_. The extract was then centrifuged at 12,000 rpm for 20 minutes, and the resulting supernatant was used for enzymatic assay. For POD activity assays, the supernatant was added to a 3 mL reaction mixture comprising 2% guaiacol, 30% H_2_O_2_, and 100 mol/L sodium phosphate buffer (pH 7.8). The reaction was incubated at 30°C for 3 minutes, terminated by adding 10 mL of 6 N hydrochloric acid. POD activity was quantified by measuring the increase in absorbance at 470 nm. SOD activity was assessed using a commercial assay kit (ab65354, Abcam) by monitoring absorbance at 450 nm, while Catalase (CAT) activity was measured with a kit (R22073-100T, Yuanye Cor, China), with one unit of CAT activity being defined as a 0.01 absorbance change at 240 nm per minute per mg of protein (Grilo et al., 2020). All assays were conducted in triplicate.

### 2.6 Determination of H_2_O_2_ and MDA contents in plants

The malondialdehyde (MDA) and H_2_O_2_ levels in leaves of wild-type and transgenic plants were determined following the protocol described by Huang et al. (2024). Specifically, the MDA content was qualified using a commercial kit (Plant MDA Assay Kit with TBA, YuanYe Biotechnology Corporation, Shanghai, China). Briefly, leaf tissue powder was mixed with 10% trichloroacetic acid followed by centrifugation at 4000 g for 10 minutes. The resulting supernatant was then reacted with 0.68% thiobarbituric acid (TBA), incubating at 95°C for 30 minutes, and centrifuged again at 4000 g for 10 minutes. MDA content = 6.45 × (OD532 - OD600) - 0.56 × OD450. For the measurement of H_2_O_2_ levels, approximately 2 g of the sample was homogenized in 2 mL of ice-cold acetone and centrifuged 12000×g, 4°C for 20 min. H_2_O_2_ content in the supernatant was determined by producing H_2_O_2_/TiO_2_ complex, whose absorbance was measured at 412 nm.

### 2.7 Measurement of Pb content by atomic absorption

Initially, seeds of the wild-type and transgenic *N. benthamiana* were germinated and grown for 21 days on MS agar plates. Subsequently, both wild-type and transgenic seedlings of *N. benthamiana* were transferred to a soil mixture (nutrient soil: vermiculite: peat, 1.5:1:1, V/V/V) amended with 0.5 mmol/L Pb acetate (Saman et al., 2022) and grown for 14 days under a 16 h/8 h photoperiod at 25°C/16°C day/night cycle. The nutrient soil was obtained from Stanley corporation (http://www.stanleygroup.cn/), with the soil organic matter (SOM) content being over 60%, the N+P_2_O_5_+K_2_O content being over 0.5% and pH value being from 5.0 to 7.0. The leaves and roots were separated, weighed, and dried at 105°C for 30 minutes, followed by 48 hours at 65°C. As described by Wang et al. (2022), the dried samples (0.1-0.2 g each) were then pretreated in 4 mL of HNO_3_ in an oven for 12 hours. The samples were further wet digested with a nitric acid and perchloric acid mixture (HNO_3_-HClO_4_, 3:1) at 300°C for 1 hour. All samples were diluted to a final concentration of 0.2% HNO_3_ and analyzed though Graphite Furnace atomic absorption spectrometer (Pinnacle 900T, Perkin Elmer, Waltham, MA, USA). Each experimental line was tested in triplicate, with each replicate comprising sample from 8-10 plants.

### 2.8 Comparative transcriptome on the transgenic and wild-type plants

After the wild-type and transgenic plants underwent the same treatment described in Section 2.8, the plant samples were stored at -80°C. Total RNA was subsequently extracted from leaves of independent transgenic (OE2 and OE10 were mixed) and wild-type lines. RNA sequencing was conducted by Sangon Biotech on the Illumina HiSeq™ platform. Following the removal of low-quality reads with Q-values below 20, clean reads were obtained. These high-quality reads were then aligned to the *N. benthamiana* “V1.0.1” reference genome (https://solgenomics.net/) utilizing HISAT2 (Kim et al., 2015). Transcript abundance was estimated in terms of Mean Transcripts Per Million (MeanTPM) (Duan et al., 2019). Differential gene expression was analyzed using DESeq2 version 1.12.4 (Ghamari et al., 2022), with differentially expressed genes (DEGs) defined as those exhibiting absolute log2 fold change ≥1 and a *q*-value <0.05. Gene Ontology (GO) and Kyoto Encyclopedia of Genes and Genomes (KEGG) pathway enrichment analyses for the DEGs were performed using the R package based on the hypergeometric distribution.

### 2.9 Analysis of rhizosphere microorganism in the transgenic and wild-type plants

Rhizosphere soil adhering to the roots of transgenic (OE2 and OE10 were mixed) and wild-type plants was collected using shovels and placed into plastic containers. Each of sample consisted of approximately 15 g (fresh weight) of pooled soils. The plants sampled were the same cohort described in Section 2.8. Genomic DNA from these soils was extracted using a standard CTAB/SDS method and stored at -80°C.

Bacterial communities within the rhizosphere were characterized based on the V5-7 variable regions of the 16S rRNA gene (Aires et al., 2018), which are amplified by use of 16S-F and 16S-R primers (**Supplementary Table S1**). The resulting PCR products were purified using magnetic beads, pooled in equal proportions based on molecular weight and DNA concentrations, and the quality of the resultant cDNA library was assessed by Qubit and real-time PCR for DNA concentration and size distribution, respectively. The qualified library was then sequenced on a NovaSeq6000 (PE250) platform at Novogene Corporation (Beijing, China). Data quality was verified using Fastp software (Version 0.23.1) (Bokulich et al., 2013). Sequence analysis was conducted using the DATA module of the Quantitative Insights into Microbial Ecology program (QIIME2, version 202202) (Zahid et al., 2021), generating amplicon sequence variants (ASVs). Taxonomic annotation of bacteria was performed using the SILVA13.8 database and NCBI taxonomy, employing the QIIME2 software with the lowest common ancestor (LCA) algorithm at a confidence cutoff of 0.8-1.0, after which non-bacterial ASVs were removed. Alpha diversity metrics, including Chao1 richness estimates, observed number of operational taxonomic units (OTUs), Shannon index, Pielou’s evenness, and Good’s coverage, were calculated using QIIME2. Beta diversity analyses such as heatmap generation, principal components analysis (PCA), and UPGMA clustering were performed in R software (version 4.0.3). Spearman correlation tests and canonical correspondence analysis (CCA) were also conducted in R software. Bacterial metabolic functions were predicted using the Phylogenetic Investigation of Communities by Reconstruction of Unobserved States (PICRUSt, version 2.3.0).

### 2.10 Statistical analysis

All experimental values were presented as means ± standard deviation. The Student’s t test was used to analyze the data with a *P*-value of less than 0.05 being considered statistically significant between the two groups (Ma et al., 2025).

## 3 Results

### 3.1 Upregulation of *CtDXS1* and *CtDXR1* genes under Pb stress

**Figure 1:**
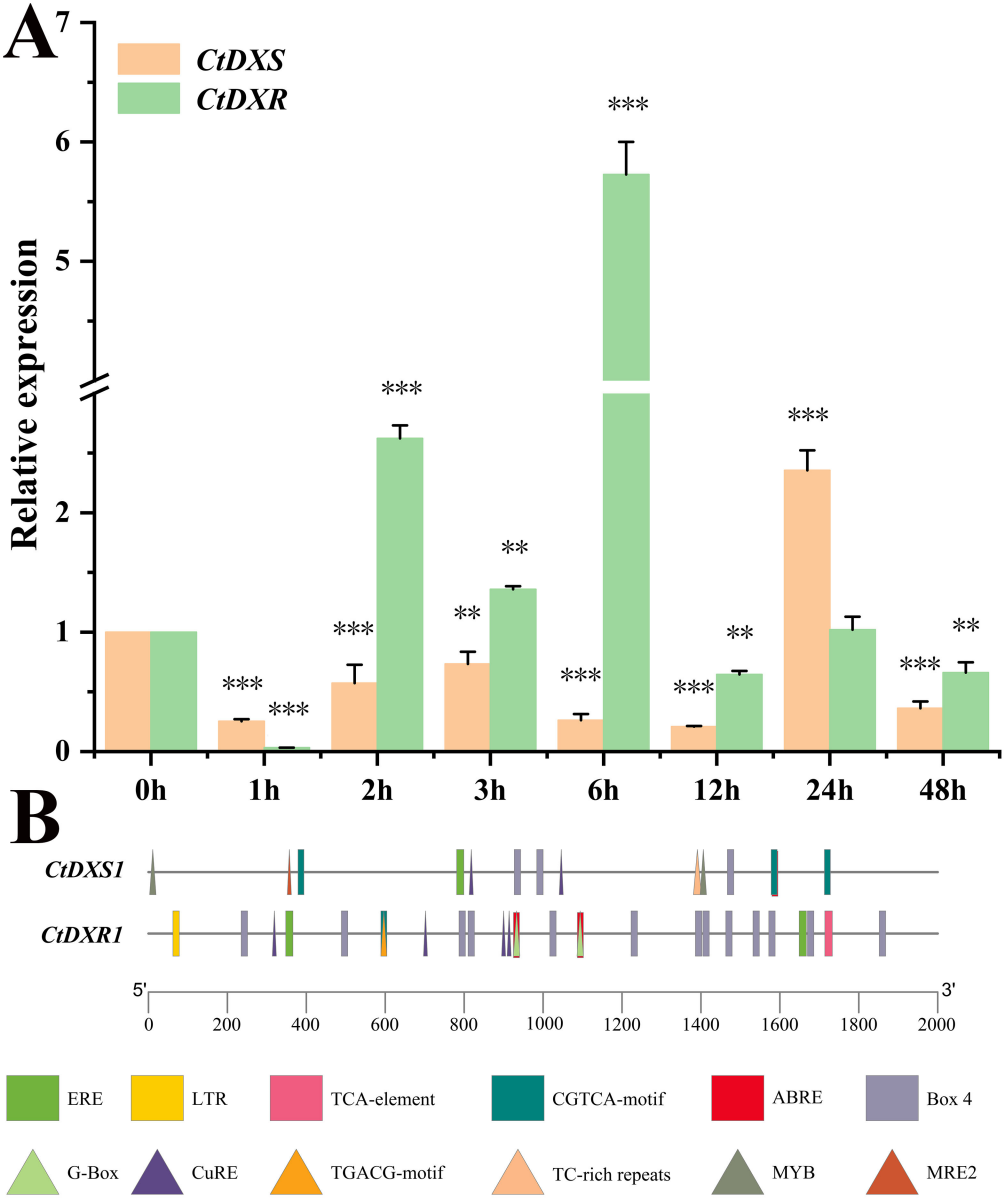
The expression patterns and *cis*-element analysis of *CtDXS1* and *CtDXR1* genes. **(A)** The real-time PCR result of *CtDXS1* and *CtDXR1* genes treated by Pb acetate. **(B)** Distribution of *cis*-elements in the promoters of *CtDXS1* and *CtDXR1 *genes. All data are mean ± SD for no less than three biological replicates. Asterisks stand for significant levels of *t*-test results of gene expression at each time point after the Pb treatment, compared to that at hour 0. *, ** and *** represent the significance difference at *P*<0.05, *P*<0.01 and *P*<0.001, respectively.

To investigate the transcriptional response of the *CtDXS1* and *CtDXR1* genes to Pb stress, their expressional levels in leaves of *C. tora* plants treated with Pb acetate were qualified using real-time PCR. Both genes revealed rapidly upregulation upon Pb exposure. Specifically, the mRNA level of the *CtDXS1* gene increased, reaching a peak of 2.36-fold after 24 hours, whereas the *CtDXR1* gene peaked earlier, reaching a maximum induction of 5.73-fold at 6 hours (**Figure 1A**). Besides, the transcription response of *CtDXS1* and *CtDXR1* genes under long-term treatment of Pb stress for 7 days was investigated. As shown in **Supplementary Figure S3**, transcripts of *CtDXS1* and *CtDXR1* genes were 4.42- and 8.94-fold higher relative to control levels (0 d) under Pb stress, however, their transcripts showed no variation between 0 d and 7 d under normal condition.

Since transcriptional regulation is largely governed by the *cis*-elements within its promoter, we analyzed the specific *cis*-elements in the promoters of the *CtDXS1* and *CtDXR1* genes using PlantCare (http://bioinformatics.psb.ugent.be/webtools/plantcare/html/) complemented by manual search. There were three types of metal-responsive elements, MRE1 (MRE, 5`-TGCRCNC-3`, R: A or G, N: any base), MRE2 (5′-HTHNNGCTGD-3′, H: A or C or T, N: any base, D: A or G or T) (metal-response element 2) and CuRE (5′-GTAC-3′) (copper response element, Zou et al., 2021), implicated to heavy metal stress. Notably, Both MRE2 and CuRE were found in the promoter of *CtDXS1* gene, whereas only CuRE was detected in the promoter of *CtDXR1* gene (**Figure 1B**). Moreover, the ABRE (ACGTG) motif, associated with ABA response, was also presented. Given previous report of Pb-induced ABA accumulation in plants (López-Orenes et al., 2020), these results suggest that the induction of *CTDXS1* and *CtDXR1* genes may occur through direct metal-responsive and (or) ABA-mediated pathway.

### 3.2 Production of *CtDXS1*_*CtDXR1* transgenic plants

The Pb-induced expression of the *CtDXS1* and *CtDXR1* genes suggests that their overexpression may enhance tolerance to Pb stress. To elucidate the functional impact of their co-expression, the modified pCambia1301 vector, carrying two independent promoters, was successfully applied to construct recombinant plasmid. Then the construct was introduced into *Agrobacterium* strain GV3101 and subsequently transformed into *N. benthamiana* (Tian et al., 2023). Among the transgenic lines, OE10 and OE2 containing low-copy insertions (data not shown) were selected, with their PCR verification at DNA level being provided in **Supplementary Figure S4**. Subsequently, the expression levels of heterologous genes were evaluated by real-time PCR. Within the line OE2, transcript levels of *CtDXS1* and *CtDXR1* were 26.36- and 48.85-fold higher, respectively, than that of the reference gene. In line OE10, these levels were 9.62- and 5.70-fold higher, respectively, than that of the reference gene (**Supplementary Figure S5**).

### 3.3 Enhanced Pb stress tolerance in transgenic plants

Seeds of transgenic (OE2 and OE10) and wild-type *N. benthamiana* were germinated on 1/2 MS medium. After 21 days, the seedlings were transplanted into soil supplemented with Pb acetate and grown for an additional 14 days. Under normal conditions, transgenic plants revealed significant morphologic advantage over wild-type plants, including longer roots (OE2), wider (OE2 and OE10) and longer leaves (OE2). However, plant height and number of leaves were not significantly different.

**Figure 2:**
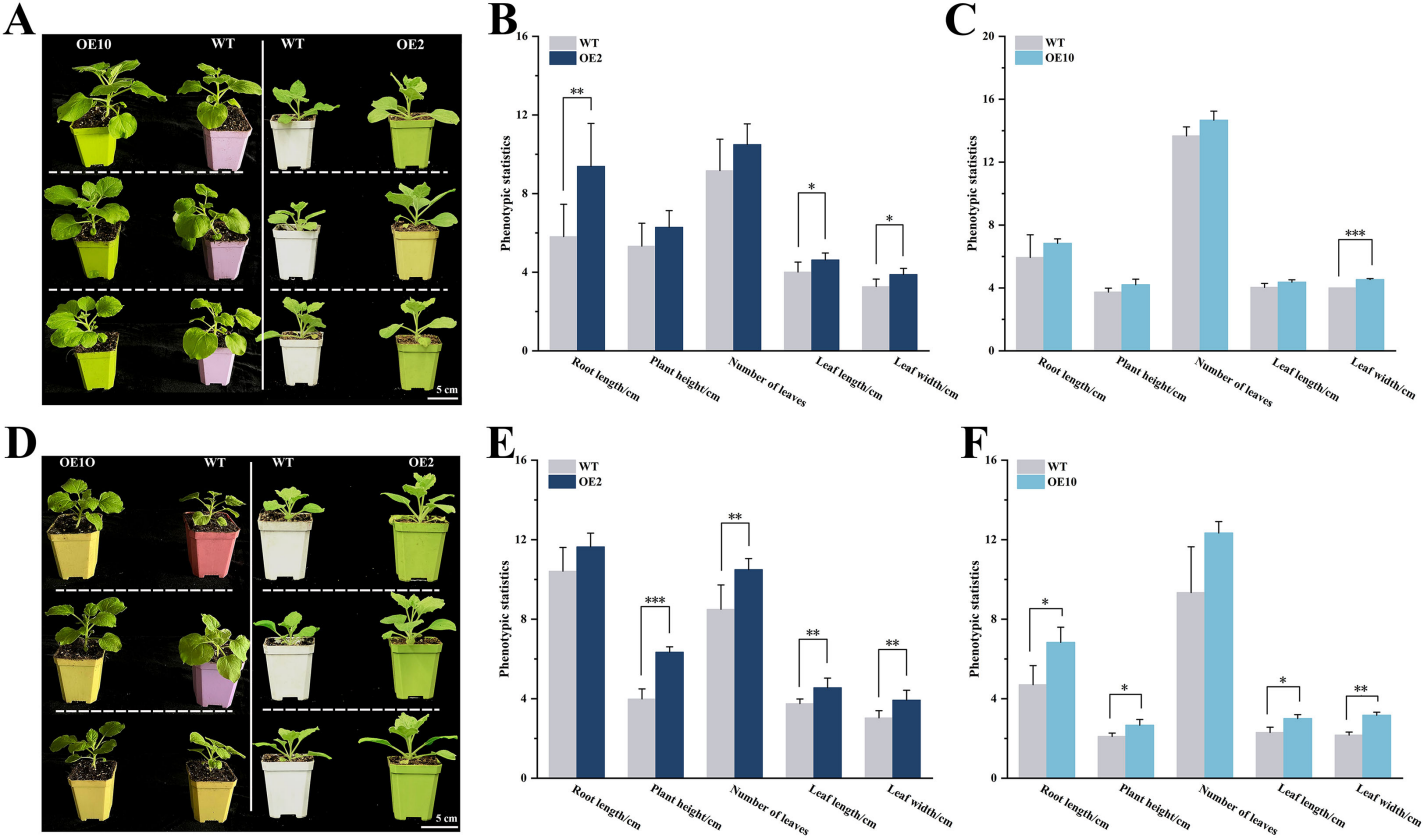
Phenotypic characterization of *CtDXS1_CtDXR1* transgenic and wild-type plants. **(A)** Photography of *CtDXS1_CtDXR1* transgenic and wild-type plants under normal conditions; **(B, C)** Phenotypic statistics of wild-type vs OE2 and OE10 under normal conditions; **(D)** Photography of *CtDXS1_CtDXR1* transgenic and wild-type plants under Pb stress; **(E, F)** Phenotypic statistics of wild-type vs OE2 and OE10 under Pb stress. Data are presented as mean ± SD of no less than three biological replicates. *, ** and *** represent the significance difference at *P*<0.05, *P*<0.01 and *P*<0.001 between wild-type and transgenic plants, respectively. The root length, plant height, leaf length and leaf width adopt “centimeter” as unit on the vertical axis, while number of leaves adopts “quantity” as unit on the vertical axis in **(B, C, E, F)**.

Interestingly, the *CtDXS1*_*CtDXR1* transgenic plants markedly enhanced tolerance to Pb stress. Compared to wild-type plants, transgenic plants showed significantly increased root length (1.12~1.45 fold), leaf width (1.29~1.46 fold), leaf length (1.22~1.30 fold), number of leaves (1.24~1.32 fold), and plant height (1.27~1.59 fold) under Pb stress (**Figure 2**). Furthermore, transgenic plants experienced less severe reduction in biomass under Pb stress. For example, Pb stress led to reductions of 0%, 36.43%, 15.96%, and 31.46% in root length, plant height, number of leaves, leaf width, and leaf length in transgenic plants, respectively, whereas those in wild-type plants were 20.74%, 43.70%, 31.75%, and 42.93%, respectively.

### 3.4 *CtDXS1_CtDXR1* transgenic plants increased isoprenoid content

**Figure 3:**
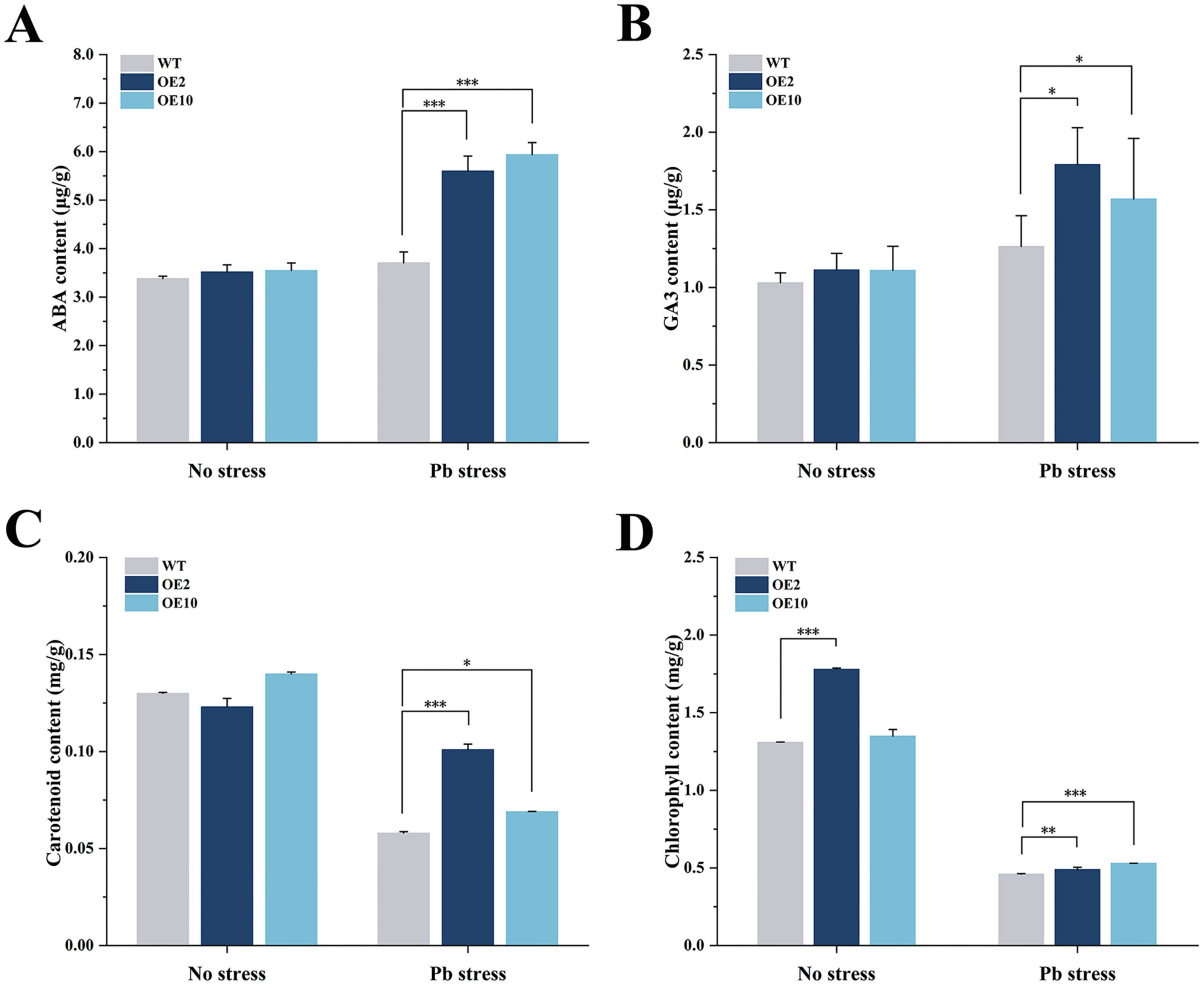
Analysis of isoprenoid content in *CtDXS1*_*CtDXR1* transgenic and wild-type plants. **(A)** ABA content; **(B)** GA3 content; **(C)** carotenoid content; **(D)** chlorophyll content. Data are represented as mean ± SD of no less than three biological replicates. *, ** and *** represent the significance difference at *P*<0.05, *P*<0.01 and *P*<0.001 between wild-type and transgenic plants, respectively.

The contents of four kinds of plastidic isoprenoids, such as ABA, GA_3_, carotenoid and chlorophyll, were detected. Given that a major portion of ABA biosynthesis is carried out via the plastidial MEP pathway, coexpression of *CtDXS1* and *CtDXR1* may affect ABA accumulation. It was observed that transgenic plants accumulated 1.51~1.60-fold of ABA compared to wild type under Pb stress (**Figure 3A**). Similarly, GA_3_, a phytohormone playing a central role in plant development, was elevated in transgenic plants, reaching 1.24~1.42-fold of the wild-type levels under Pb stress (**Figure 3B**). The transgenic plants also exhibited a 1.19~1.74-fold of carotenoid compared with wild-type plants under Pb stress (**Figure 3C**), along with a moderate but significant increase in total chlorophyll content, ranging from 1.07~1.15-fold of wild-type levels under Pb stress (**Figure 3D**).

### 3.5 ABA played role in tolerance to Pb stress

**Figure 4:**
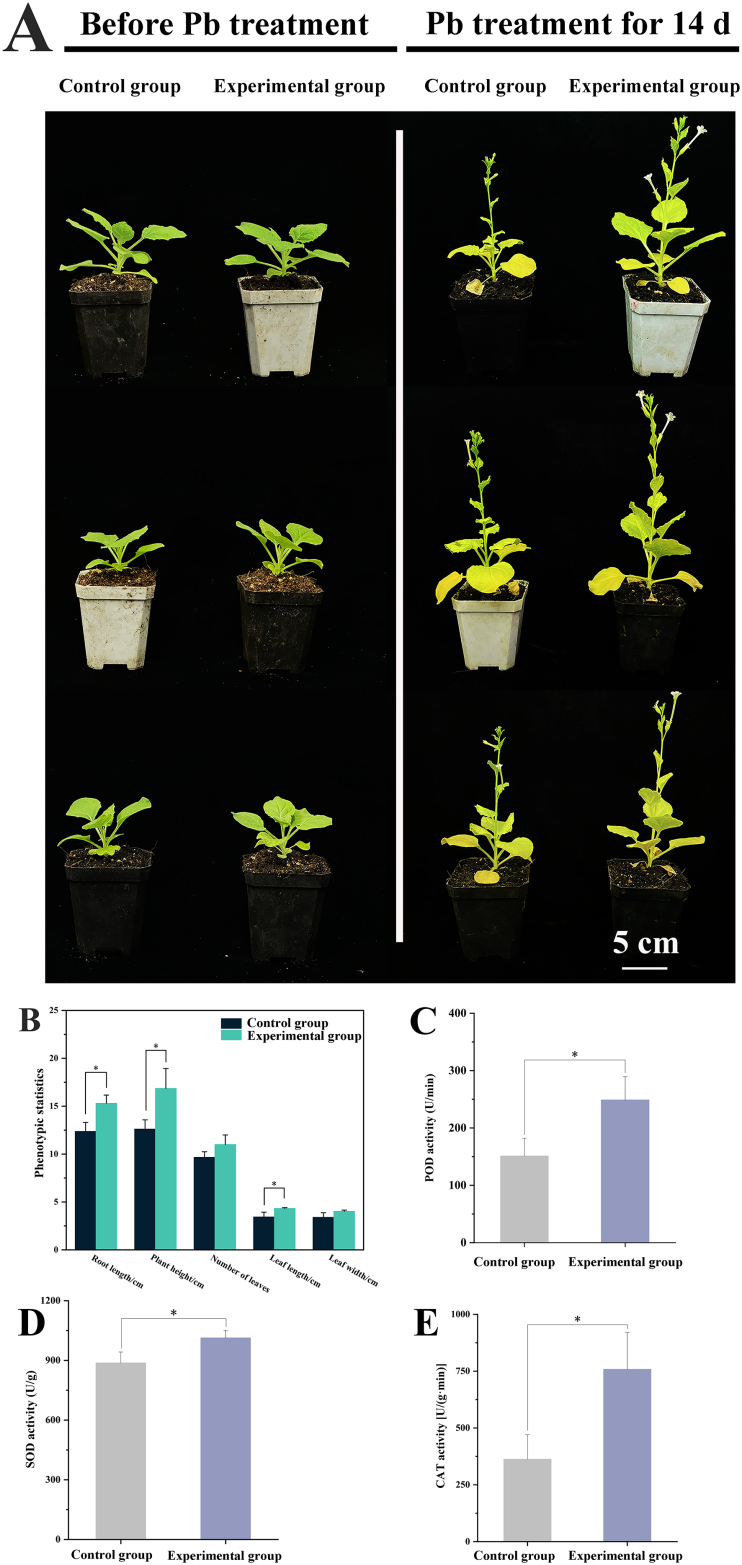
Effect of exogenous ABA on wild-type *N. benthamiana* plants under Pb stress. The 21-day-old seedlings were randomly divided into two groups (control and experimental group) followed by cultivation in a soil mixture containing 0.5 mmol/L Pb acetate. Compared with those in control group, plants in experimental group were additionally sprayed with 100 µmol/L of ABA solution. **(A)** The photos of plants before and after Pb treatment; **(B)** The phenotypic statistics of plants. The root length, plant height, leaf length and leaf width adopt “centimeter” as unit on the vertical axis, while number of leaves adopts “quantity” as unit on the vertical axis; **(C)** POD activities; **(D)** SOD activities; **(E)** CAT activities. Data are presented as mean ± SD of no less than three biological replicates. * represents the significance difference at *P*<0.05 between experiment group and control group.

To evaluate whether ABA contributed to Pb stress tolerance, 100 μmol/L of ABA was exogenously applied to wild-type plants. As a result, exogenous ABA application significantly improved phenotypic performance under Pb stress (**Figure 4A**). After 14 days of Pb treatment, the ABA-treated plants showed increased plant height (16.85 cm; *p*<0.05), leaf number (11.00), root length (15.31 cm, *p*<0.05), leaf length (4.33 cm, *p*<0.05), and leaf width (4.03 cm) compared to untreated controls (12.61 cm, 9.67, 12.37 cm, 3.44 cm and 3.40 cm), respectively (**Figure 4B**).

Biochemical assays displayed that exogenous ABA led to enhancement in the activities of key antioxidant enzymes (**Figures 4C-E**). The activities of POD, SOD, and CAT were elevated by 1.65 times (*P*<0.05), 1.14 times (*P*<0.05) and 1.50 times (*P*<0.05) higher than those in the control group, respectively. These results highlight that ABA strengthens the antioxidant enzyme defense system, potentially migrating Pb-induced oxidative damage.

### 3.6 Coexpression of *CtDXS1* and *CtDXR1* genes increased antioxidant enzyme activities

**Figure 5:**
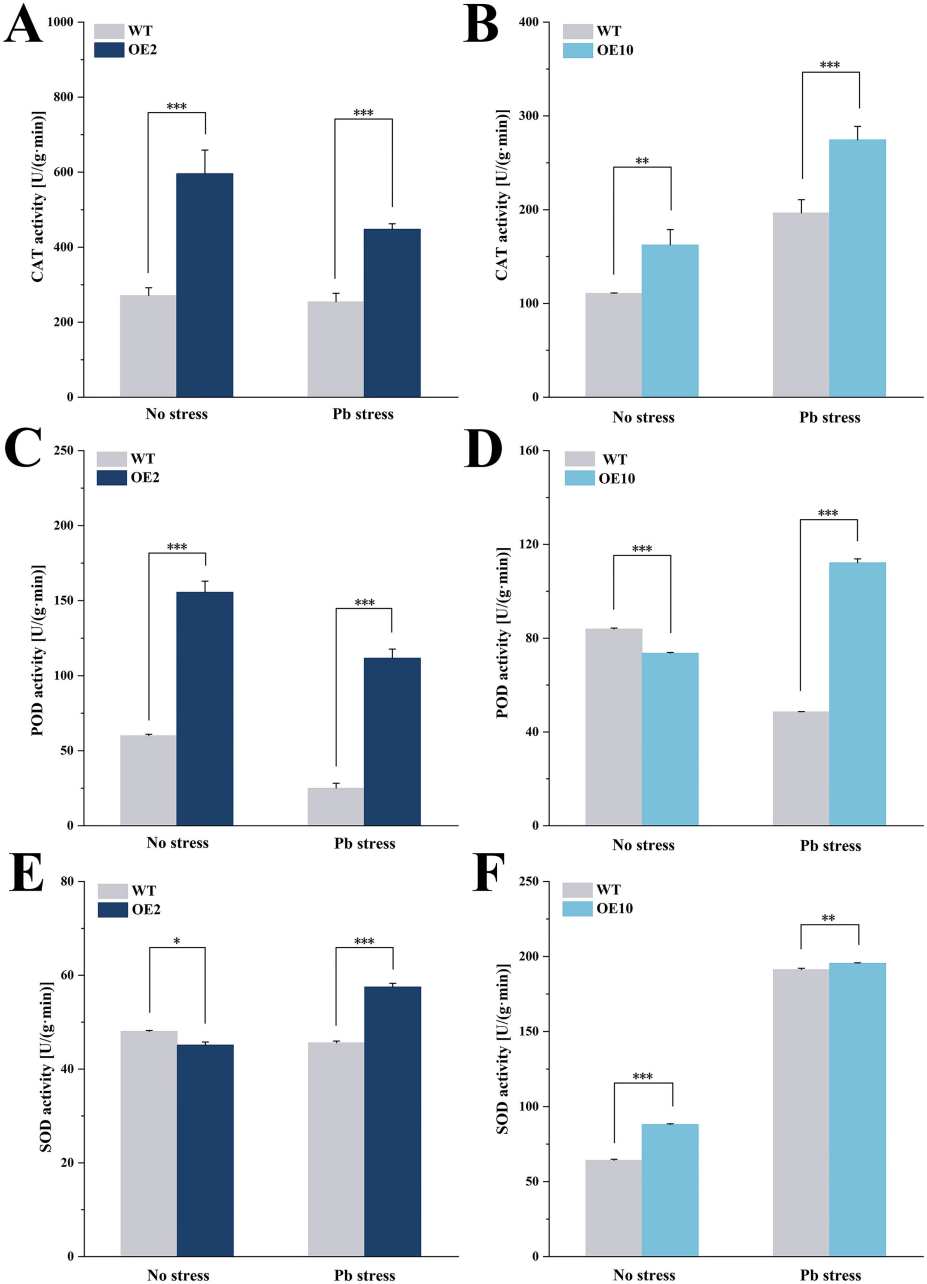
Antioxidant enzyme activities in *CtDXS1*_*CtDXR1* transgenic and wild-type plants under normal condition and Pb treatment. **(A)** CAT activities in wild-type and OE2 plants; **(B)** CAT activities in wild-type and OE10 plants; **(C)** POD activities in wild-type and OE2 plants; **(D)** POD activities in wild-type and OE10 plants; **(E)** SOD activities in wild-type and OE2 plants, and **(F)** SOD activities in wild-type and OE10 plants. Data are presented as mean ± SD of three biological replicates. *, ** and *** represent the significance difference at *P*<0.05, *P*<0.01, and *P*<0.001 between wild-type and transgenic plants, respectively.

As shown in **Figures 5A, B**, overexpression of *CtDXS1* and *CtDXR1* genes resulted in significant increase in CAT activity. Under normal conditions and Pb stress, total CAT activity in transgenic plants was 1.56~2.39-fold and 1.50~1.83-fold of that in wild-type plants, respectively. Although the POD activity was lower in transgenics under normal conditions, it was notably elevated after Pb treatment, reaching 2.31~2.36-fold higher relative to wild-type level (**Figures 5C, D**). In addition, the relatively enhanced SOD activity was observed in transgenic plants, with 1.02~1.26-fold of that in wild-type plants (**Figures 5E, F**).

### 3.7 Coexpression of *CtDXS1* and *CtDXR1* genes decreased H_2_O_2,_ MDA and Pb contents

**Figure 6:**
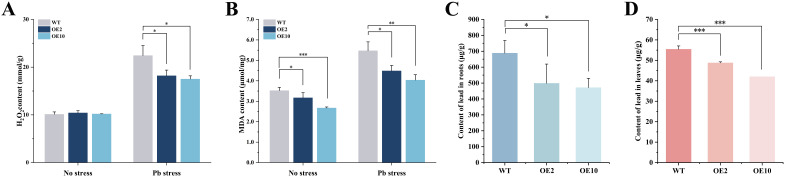
Contents of H_2_O_2_, MDA, and Pb in *CtDXS1*_*CtDXR1* transgenic and wild-type plants under normal and Pb-stress conditions. **(A)** H_2_O_2_ content; **(B)** MDA content; **(C)** Pb content in the roots; **(D)** Pb content in the leaves. Data are presented as mean ± SD of three biological replicates. *, ** and *** represent the significance difference at *P*<0.05, *P*<0.01 and *P*<0.001 between wild-type and transgenic plants, respectively.

Both H_2_O_2_ and MDA serve as indices of oxidative damage and lipid peroxidation under adverse conditions, which in turn trigger the antioxidant defense system. Under Pb stress, wild-type plants induced higher H_2_O_2_ accumulation, whose content increased to 2.22-fold compared to that under normal condition, indicating substantial oxidative damage occurred in response to Pb stress. In contrast, the transgenic plants accumulated only 78.06~81.18% of H_2_O_2_ levels in respect to wild-type levels under Pb stress (**Figure 6A**).

A similar changing tendency was observed in MDA accumulation. In wild-type plants, Pb stresses resulted in 1.54-fold level of MDA content relative to that under normal conditions. Intriguingly, transgenic plants accumulated merely 74.22~81.99% of MDA content as compared with wild-type plants under Pb stress. The concurrent reduction in H_2_O_2_ and MDA levels coupled with enhanced antioxidant enzyme activities indicate that the decreased oxidative damage in *CtDXS1*_*CtDXR1* transgenic plants is mediated by reinforcing antioxidant enzyme system (**Figure 6B**).

Pb is preliminarily absorbed by plants as a divalent cation (Pb 2^+^), whose absorption initiates the adsorption of the metal ion to the root surface followed by root uptake. After 14 days of Pb treatment, the transgenic plants accumulated significantly less Pb in both roots and leaves compared to wild-type plants. Pb concentrations in transgenic roots and leaves were 68.50~72.41% and 75.77~87.98% of those in wild-type levels, respectively (**Figures 6C, D**). The evidence indicates that overexpression of *CtDXS1* and *CtDXR1* genes limits the Pb uptake and accumulation in plants.

### 3.8 Transcriptomic alteration in *CtDXS1_CtDXR1* transgenic plants under Pb stress

**Figure 7:**
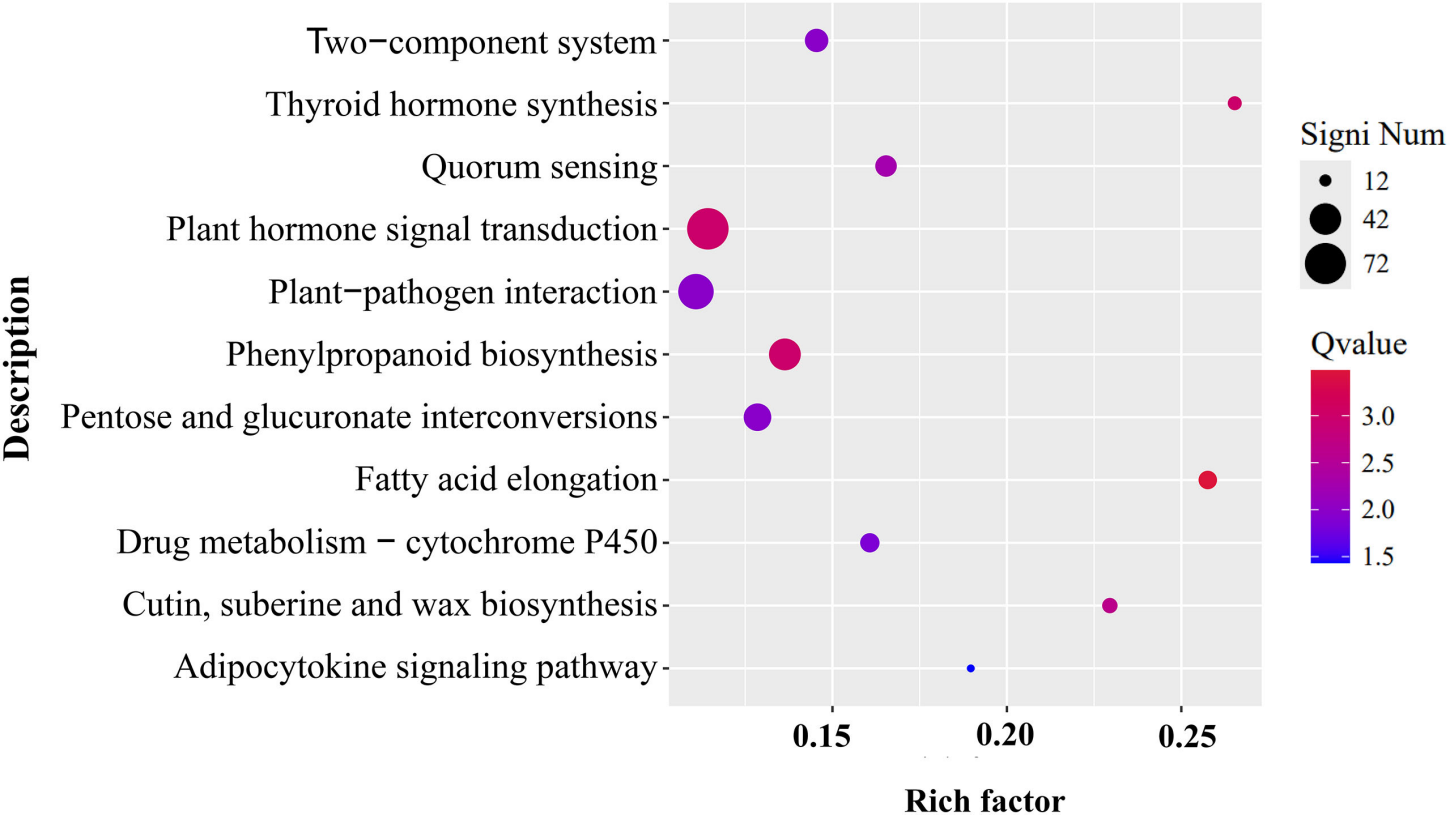
KEGG analysis of up-regulated DEGs in *CtDXS1*_*CtDXR1* transgenic plants. Only the most significant enriched KEGG pathways with Q-value <0.05 are presented.

Given that co-expression of *CtDXS1* and *CtDXR1* genes not only enhanced ABA accumulation, but also conferred Pb tolerance, it became crucial to explore the potential underling mechanisms through transcriptomic profiling. Two cDNA libraries were constructed from wild-type and transgenic plants under Pb stress. RNA-seq generated more than 59.93 and 46.83 M clean reads for wild-type and transgenic library, respectively. Of these, 91.21%-93.62% were uniquely mapped to the *N. benthamiana* genome, with Q30 quality score exceeding 95.82%, confirming the reliability of transcriptome data (**Supplementary Table S2**). In respect to wild-type plants, 1,545 down-regulated and 5,456 up-regulated genes were found in the transgenic plants, indicating broad transcriptomic reprogramming in transgenic plants. KEGG enrichment pathway analysis of up-regulated genes revealed 11 significantly enriched pathways in transgenic plants (**Figure 7**; **Supplementary Table S3**). The top three pathways were “Plant hormone signal transduction,” “Plant-pathogen interaction,” and “Phenylpropanoid biosynthesis,” accounting for 72, 52, and 42 genes respectively, with details provided in **Supplementary Table S3**. Enrichment network analysis further cluster these pathways into functional groups. One included fatty acid elongation/plant-pathogen interaction/plant hormone signal transduction/thyroid hormone synthesis, while another encompassed quorum sensing/pentose and glucuronate interconversions/two-component system (**Supplementary Figure S6**). Prior studies have demonstrated that fatty acid, phenylpropanoid-derived metabolites, and pentose and glucuronate interconversions genes were positively correlated with heavy metal tolerance (Dong et al., 2023; Sun et al., 2020; Wang et al., 2013), and quorum sensing has been shown to improve the stability and adaptability of the rhizosphere microbial community (Hao et al., 2022).

### 3.9 Real-time PCR verification

**Figure 8:**
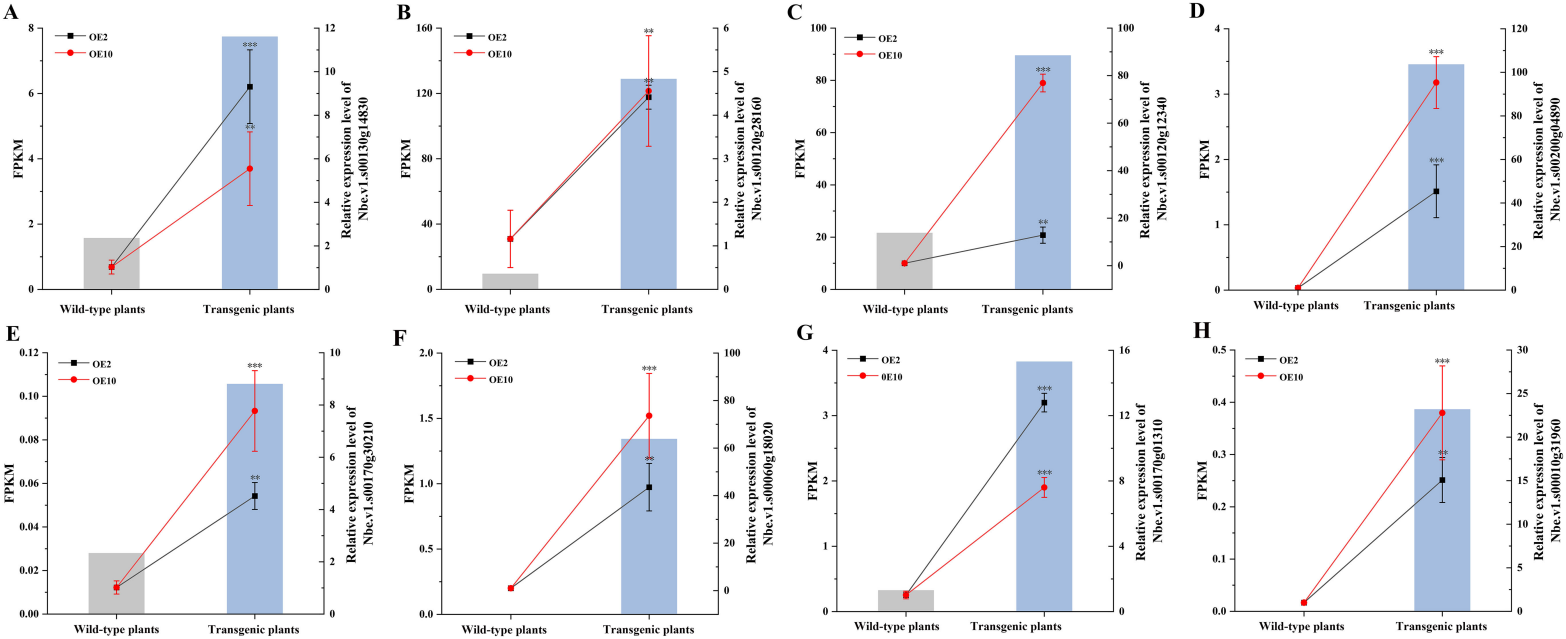
Real-time PCR verification of eight putative Pb-tolerant genes in *CtDXS1*_*CtDXR1* transgenic and wild-type plants under Pb treatment. The left vertical axis indicates FPKM values from transcriptome while the right vertical axis indicates fold change as measured by real-time PCR. **(A)** Nbe.v1.s00130g14830; **(B)** Nbe.v1.s00120g28160; **(C)** Nbe.v1.s00120g12340; **(D)** Nbe.v1.s00200g04890; **(E)** Nbe.v1.s00170g30210; **(F)** Nbe.v1.s00060g18020; **(G)** Nbe.v1.s00010g31960; **(H)** Nbe.v1.s00170g01310. Data are presented as mean ± SD of no less than three biological replicates. ** and *** represent the significance difference at *P*<0.01 and *P*<0.001 between transgenic and wild-type plants, respectively.

Eight DEGs implicated in heavy metal tolerance were selected for real-time PCR verification, with *N. benthamiana EF-1α6* gene being as reference gene (NbEF-1α-F and NbEF-1α-R as primers, Chi et al., 2019). These included one mitochondrial transcription termination factor (mTERF) (Nbe.v1.s00170g01310), three ABC transporter (Nbe.v1.s00120g12340, Nbe.v1.s00010g31960 and Nbe.v1.s00170g30210), two ATPase (Nbe.v1.s00200g04890, and Nbe.v1.s00060g18020), and two cysteine proteinase Cathepsin (Nbe.v1.s00130g14830, and Nbe.v1.s00120g28160). All eight DEGs exhibited relatively higher expression levels in the transgenic plants, consistent with the transcriptomic profiling results (**Figure 8**).

### 3.10 PPI network analysis

**Figure 9:**
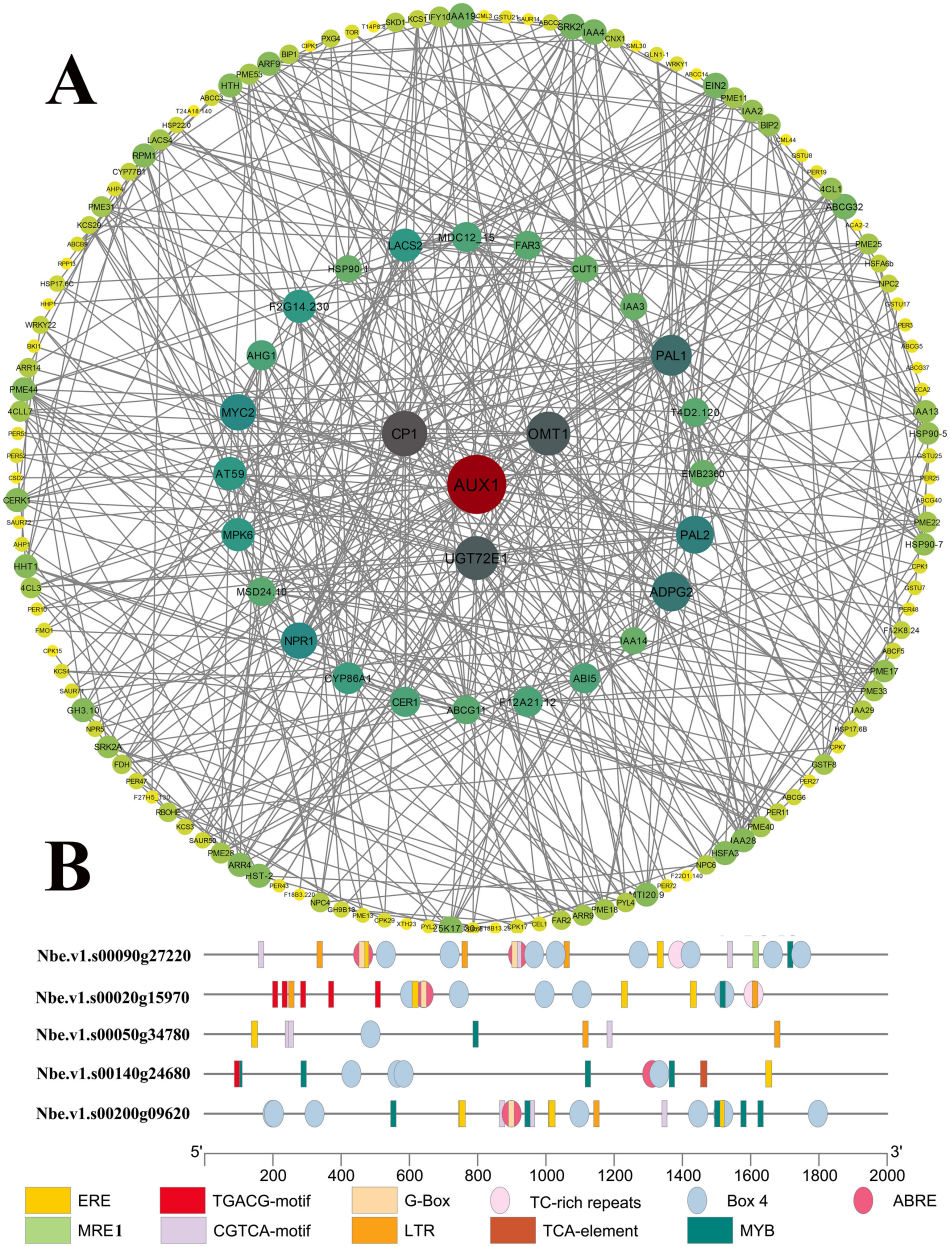
The Pb-tolerant network in *CtDXS1*_*CtDXR1* transgenic plants based on the protein-protein interactome prediction. **(A)** PPI analysis; **(B)***Cis*-acting element analysis on the four core genes.

Beyond the enriched pathways potentially linked to heavy metal stress tolerance, several functional protein families, including ATPases, ABC transporters, heat shock transcription factors (Hsf), small heat shock proteins (sHsp), antioxidant enzymes, and glutathione S-transferases (GST), have been previously reported to be associated with heavy metal tolerance in plants (Wang et al., 2021). Thus, it is logical to highlight candidate downstream players in Pb tolerance by comparing their expression between wild-type and transgenic plants. Totally, 389 unrepeated genes exhibited higher expression levels in transgenic plants compared with those in wild-type plants, including 26 ATPases, 18 ABC transporters, one metallothionein-like proteins, two Hsfs, eight Hsp70, eight sHsps, two SODs, eight GST genes and 316 genes from the 11 enriched KEGG pathways. Subsequently, 202 members revealed interaction in the protein-protein interactome analysis using the STRING database (http://cn.string-db.org, Zhang et al., 2024c). As shown in **Figure 9** and **Supplementary Table S4**, the resulting network comprised 202 nodes (genes) interacting with 1,289 neighbors through 644 edges, with an average number of neighbors of 6.38 and an average local clustering coefficient of 0.5. The PPI enrichment *p*-value was less than 1.0e-16. Based on the node connectivity, the top four core proteins were Auxin transporter protein 1 (AUX1, 32 interactions), Calcium-binding protein CP1 (CP1, 23 interactions), Flavone 3’-O-methyltransferase 1 (OMT1, 22 interactions) and UDP-glycosyltransferase 72E1 (UGT72E1, 22 interactions). AUX1 facilitates polar transportation and is involved in plant growth and development, root-soil metabolites exchange, and heavy metal response (Yu et al., 2015). CP1 (Ca^2+^ binding proteins) are core members of the calcium-mediated ABA signaling pathway under abiotic and biotic stress (Jing et al., 2016; Takahashi et al., 2000). OMTs are responsible for the biosynthesis of stress-related plant natural products (Huang et al., 2024; Lee et al., 2017) and is ABA-inducible (Giordano et al., 2016). UGTs function as detoxification enzymes and glycosylates lipids, hormones, and secondary metabolites (Shen et al., 2025). The expression of UGTs is upregulated under ABA treatment and their overexpression is used to improve abiotic stress tolerance (Zhang et al., 2021). These core proteins interacted with transcription factors related to a variety of plant hormone signaling pathways (e.g., Ethylene signaling pathway, MAPK signaling pathway, jasmonic acid signaling pathway and ubiquitin signaling pathway), ABC transporters, and enzymes related to phenylpropanoid biosynthesis, which provides insights into the intricate crosstalk among these pathways (**Figure 9A**). In addition, the ABC transporters exhibited close connections with each other, implying their coordinate roles in plant hormone signaling pathways and heavy metal translocation. Meanwhile, the complex interactions among transcription factors might provide multilevel supervision on Pb tolerance and plant growth (**Supplementary Table S4**). For instance, AUX1 and CP1 showed not only interaction with each other but also interaction with other nodes simultaneously, such as transcription factor MYC2 (MYC2) and Mitogen-activated protein kinase 6 (MPK6). MYC2 functions as transcriptional activators in ABA-mediated signaling by interacting specifically with the promoter of responsive to dehydration 22 (RD22) gene (Abe et al., 2003). MPK3 and MPK 6 are core members of the MAPK family that interacts with the ABA signaling pathway, participating in the response to abiotic and biotic stress (Manna et al., 2023). The enzymes related to phenylpropanoid biosynthesis also revealed close and complex interactions, which might provide a subtle regulation on the phenylpropanoid biosynthesis (**Supplementary Table S4**). AUX1 and CP1 also interacted with protein phosphatase type 2C (PP2C), which are involved in ABA metabolism. Given that AUX1 and CP1 are closely associated with the ABA-mediated signaling pathway, it is reasonable to consider both members as core regulators in Pb tolerance.

Furthermore, *cis*-acting element analysis on promoters of these core genes revealed at least one ABRE element in AUX1 (Nbe.v1.s00140g24680), CP1 (Nbe.v1.s00020g15970), UGT72E1 (Nbe.v1.s00090g27220) and OMT1 (Nbe.v1.s00200g09620), while at least one MYB element in OMT1 (Nbe.v1.s00050g34780). ABREs and MYBs (MYB binding elements) are known to mediate ABA-dependent stress responses (Abe et al., 2003), and therefore, the increased transcript levels of these core genes are closely related to an ABA-dependent mechanism underlying enhanced Pb tolerance in *CtDXS1*_*CtDXR1* transgenic plants.

### 3.11 Sequencing statistics and diversity of bacterial communities

A total of 189,062 clean sequences were obtained from rhizosphere soil samples of wild-type plants and transgenic plants under normal and Pb-stress conditions. The number of ASVs ranged from 660 to 981, with Good’s coverage exceeding 99.6% across all samples, indicating that the sequence depth adequately covered the bacterial communities. Rarefaction curves and Chao1 values indicated that Pb stress resulted in a decrease in taxa richness, with declines of 27.1% in wild-type plants and 4.9% in transgenic plants. Interestingly, transgenic plants maintained higher Chao1 values under Pb stress compared to wild-type plants. Similarly, the Shannon index showed a significant decrease in bacterial biodiversity in wild-type plants under Pb stress, dropping by 34.03% (from 6.765 to 4.463), compared to only 5.23% (from 6.117 to 5.797) in transgenic plants. Consistently, the Pielou’s evenness index declined by 30.06% decline in wild-type plants compared to a minor decrease (4.34%) in transgenic plants under Pb stress. Overall, Pb stress had a strong inhibitory effect on community biodiversity in wild-type plants, while transgenic plants maintained greater community stability (**Supplementary Table S5**).

Taxonomic assignment of sequence tags identified 16-18 phyla, 27-37 classes, 70-93 orders, 100-140 families, and 135-188 genera. As illustrated in **Supplementary Figure S7A**, the dominant phyla across samples were *Proteobacteria*, *Cyanobacteria*, and *Actinobacteriota*, collectively accounting for over 91.53% of the classified sequences. Under normal conditions, transgenic plants hosted a higher abundance of Cyanobacteria but lower abundances of *Proteobacteria* and *Actinobacteriota* compared to wild-type plants. Under Pb stress, the bacterial communities in the rhizosphere soils of both plant types showed similar trends, albeit at different magnitudes. Wild-type plants displayed a decrease in the abundances of *Proteobacteria* (36.05%) and *Actinobacteriota* (31.41%), and an increase in *Cyanobacteria* (91.79%). In contrast, transgenic plants showed a smaller decline in the abundance of *Proteobacteria* (21.50%) and *Actinobacteriota* (2.17%), and a smaller increase in *Cyanobacteria* (26.21%). Notably, the abundances of *Bacteroidota* and *Myxococcota* in transgenic plants increased by 1.62- and 2.41-fold under Pb stress, respectively, consistent with their documented roles in water-polluted sediments (Li et al., 2023; Zhang et al., 2024b). At the genus level, the most dominant genera were unidentified_Chloroplast, Pseudomonas, and the Allorhizobium-Neorhizobium-Pararhizobium-Rhizobium (ANPR) complex, collectively accounting for 54.47% of the classified genera (**Supplementary Figure S7B**). Under normal conditions, transgenic plants exhibited a higher abundance of the ANPR complex but lower abundances of unidentified_Chloroplast and Pseudomonas compared to wild-type plants. Under Pb stress, wild-type plants showed 80.09% decrease in ANPR and 93.49% increase in unidentified_Chloroplast and 37.74% increase in Pseudomonas. Conversely, transgenic plants demonstrated an 80.76% reduction in Pseudomonas, a 26.42% increase in unidentified_Chloroplast, and a 42.17% increase in *Cyanobacteria*.

**Figure 10:**
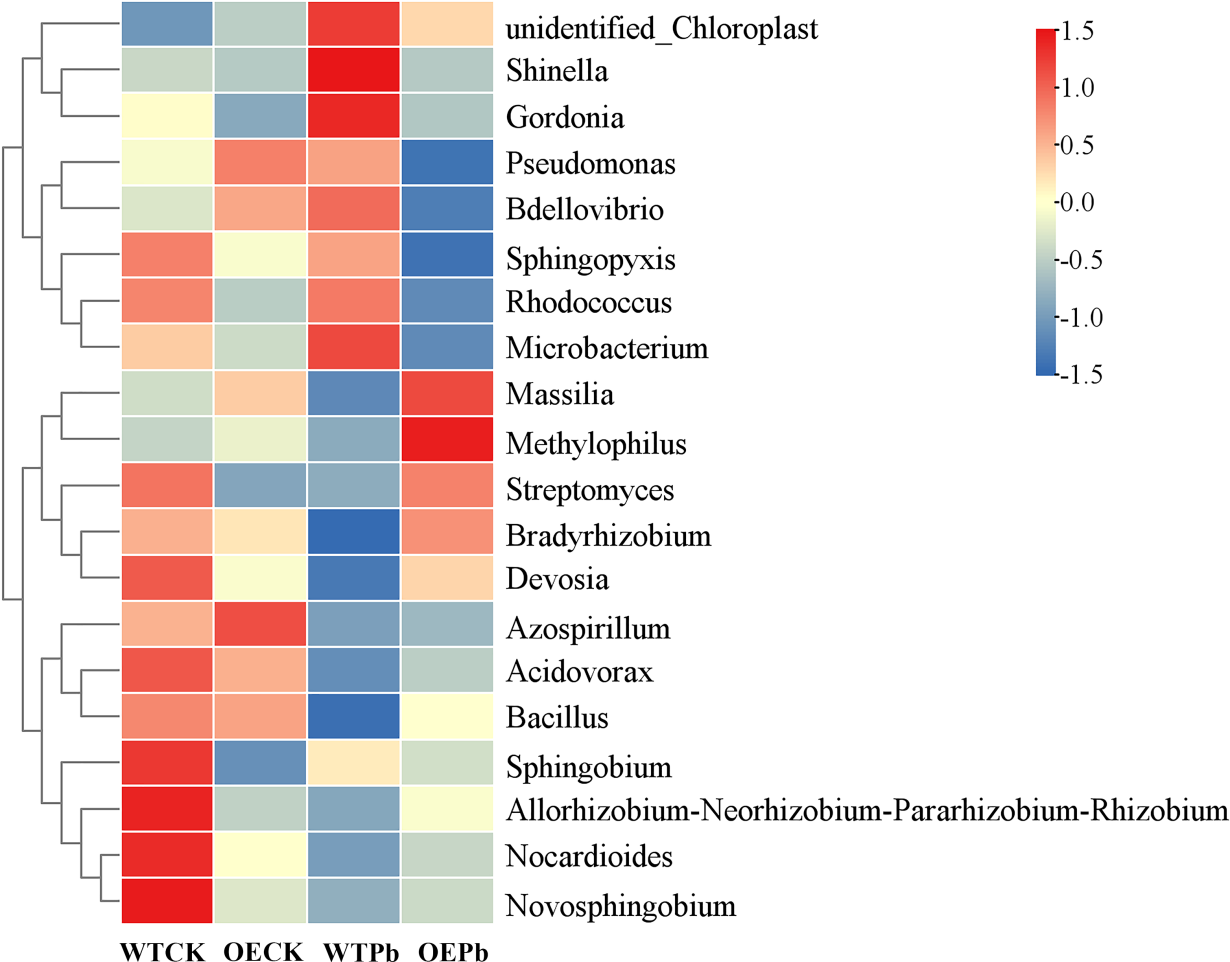
Heatmap of rhizosphere microbial community at genus level. WTCK, WTPb, OECK, and OEPb represent rhizosphere microbial samples of wild-type plants under normal condition, wild-type plants under Pb stress, transgenic plants under normal condition, and transgenic plant under Pb stress, respectively.

To further elucidate community differences associated with Pb tolerance, an abundance-based heatmap of the top 20 genera was generated. As depicted in the **Figure 10**, three genera—*Methylophilus*, *Massilia* and *Bradyrhizobium*—were not only highly abundant in transgenic plants under Pb stress but also showed the greatest abundance differences compared to wild-type plants under similar conditions. Specifically, the abundance of *Methylophilus* was 3.90-fold, 2.39-fold, and 5.71-fold higher in transgenic plants under Pb stress compared to wild-type plants under normal conditions, transgenic plants under normal conditions, and wild-type plants under Pb stress, respectively. The abundance differences for genus *Massilia* were 2.54-fold, 1.35-fold, and 5.31-fold, respectively, while those for genus *Bradyrhizobium* were 1.20-fold, 1.15-fold, and 4.43-fold, respectively. These findings are consistent with previous research, which indicated that the genus *Massilia* was enriched in contaminated soil and supported nutrient supply for *Imperata* cylindrica (Mao et al., 2024; Zhang et al., 2016). Genus *Methylophilus*, known for its plant growth-promoting traits, shows promise for application in phytoremediation (Wang et al., 2022; Zhang et al., 2019), while genus *Bradyrhizobium* has been found enriched in the rhizosphere of *Athyrium wardii*, a plant used in phytostabilization (Zhang et al., 2024d).

## 4 Discussion

Heavy metal stress poses significant threats to plant growth, development, and nutritional accumulation, thereby compromising food safety and human health. Plants tolerance to heavy metals is determined by a combination of intrinsic physiological activities and the structure-function dynamics of the soil microbial community. In the plants themselves, it is promising to enhance adaptation to Pb stress by reducing Pb uptake and mitigating the excess of ROS, since Pb stress—similar to stresses from other heavy metals like Cr and Hg—triggers excessive ROS accumulation. Within the plant defense system, ABA is particularly notable as it regulates various aspects of plant growth and development (Yu et al., 2020, 2023). The plastid-located MEP pathway operates the early stage of ABA biosynthesis. Numerous evidences have shown that genes within the MEP pathway play crucial roles in plant responses to environmental stressors like drought and in defense against pathogen infections (Tian et al., 2023; Wei et al., 2019). However, their specific function in heavy metal tolerance remains largely unexplored. To investigate the potential roles of the *CtDXS1* and *CtDXR1* genes, we first examined their expression patterns under Pb stress. Both genes exhibited upregulation upon short-term and long-term Pb exposure (**Figure 1**). Generally, induced genes are more likely to play crucial role in environmental stress tolerance compared to non-induced genes, akin to the caffeic acid O-methyltransferase gene in *Ligusticum chuanxiong* (Huang et al., 2024), the zinc-finger protein gene in soybean (Wei et al., 2023), and the aldehyde dehydrogenase gene in *Arabidopsis thaliana* (Sunkar et al., 2003). Furthermore, both rapidly and slowly responsive genes are implicated in plant adaption to environmental stress such as heavy metal and drought stress. For instance, Li et al. (2017) found that several terpenoid-biosynthetic unigenes encoding DXS, *ent*-kaurene synthase, and *ent*-kaurene oxidase were significantly induced in *Festuca arundinacea* after 4 h of Pb treatment. In addition, the rapidly induced CAT gene in *Festuca arundinacea* (Li et al., 2017) would result in increasing antioxidant ability and reduced level of oxidative damage caused by Pb toxicity. Conversely, long-term Pb treatment (6 days) in grass pea led to enhanced activities of SOD and POD, along with the induction of phytochelatin synthase gene (Abdelkrim et al., 2023). Phytochelatin synthase gene has been demonstrated to confer Cd tolerance (Zhu et al., 2021). Hence, both genes were hypothesized to be potentially correlated with Pb stress response, and this hypothesis was confirmed by further transgenic experiments.

As shown in **Figure 2**, the *CtDXS1*_*CtDXR1* transgenic plants displayed greater biomass than the wild-type plants under normal conditions, suggesting that overexpression of the *CtDXS1* and *CtDXR1* genes promotes plant growth. More importantly, the *CtDXS1*_*CtDXR1* transgenic plants demonstrated superior tolerance to Pb stress than wild-type plants, indicating that enhancement of Pb tolerance can be achieved without compromising growth. Similar trade-offs between plant growth and stress tolerance have also been reported for VuNAC1/2 transcription factors in cowpea (Srivastava et al., 2022) and in transgenic plants co-overexpressing dehydration responsive element binding protein (DREB) and phytochrome-interacting factor (PIF) transcription factor (Kudo et al., 2017). As the overexpression of *CtDXS1* and *CtDXR1* enhanced Pb tolerance, their increased transcripts in response to Pb stress suggests that both genes may function as positive regulator of the plant responses to Pb stress.

The four key types of plastidic isoprenoids—carotenoids, chlorophyll, GA3, and ABA—are derived from the common intermediate geranylgeranyl pyrophosphate (GGPP) through three pathways (**Supplementary Figure S1**). Although the levels of these isoprenoids varied complexly under various conditions, *CtDXS1*_*CtDXR1* transgenic plants accumulated higher levels of all four isoprenoids compared to wild-type plants in Pb-contaminated soil. Among these, GA3 was a key regulator of plant growth, as *CtDXS1*_*CtDXR1* transgenic plants exhibit relatively higher GA3 content than wild-type plants under normal condition. The increased accumulation of chlorophyll in *CtDXS1*_*CtDXR1* transgenic plants under Pb stress could be attributed to the elevated ABA content, which is known to protect chlorophyll pigments under abiotic stress (Huang et al., 2017; Xu et al., 2022). Interestingly, the relative increase in carotenoids was more limited than that in ABA. A similar disparate was observed by Estévez et al. (2001) in *DXS*-overexpressing *Arabidopsis*, where change in ABA content was greater than that of total carotenoid content, potentially due to the complex ABA metabolism involving process such as reactivation from glucose-conjugated form by *β*-glucosidase (Han et al., 2020b). The results presented in **Figure 4** provided evidence that ABA played substantial role in plant tolerance to Pb stress. Considering that *CtDXS1*_*CtDXR1* transgenic plants showed similar trends of increased ABA, ABA might act alone or be of more significance than other isoprenoids under Pb stress. Thus, these results suggest that the *CtDXS1* and *CtDXR1* genes likely influence ABA accumulation directly or indirectly, thereby enhancing tolerance to Pb stress.

Pb stress triggers oxidative damage, underscoring the importance of ROS detoxification for heavy metal tolerance. Key ROS-scavenging enzymes, primarily consisting of SODs, PODs, and CATs, are central to cellular defense. Exogenous ABA application also significantly reduced heavy metal content in plants and heavy metal influx among various tissues, as demonstrated in Pingyi sweet tea (Deng et al., 2021) and poplar (Shi et al., 2019). Moreover, recent studies have shown that overexpression of the MhNCED3 gene, a key enzyme in the late stage of ABA biosynthesis, reduced the expression of Cd^2+^-uptake-related genes (NRAMP and IRT), thereby decreasing Cd^2+^ influx and Cd content in *Arabidopsis* and apple (Zhang et al., 2019). Our results demonstrated that transgenic plants exhibited enhanced SOD, POD, and CAT activities compared to wild-type plants, mirroring the effect of exogenous ABA application (**Figure 4**). Correspondingly, MDA, H_2_O_2_, and Pb levels were significantly lower in the transgenic plants compared to wild-type plants during Pb exposure. Accordingly. we propose that co-expression of *CtDXS1*_*CtDXR1* increase the activities of these ROS-scavenging enzymes and reduce Pb uptake, potentially through endogenous ABA accumulation, thereby substantially migrating ROS toxicity and improving cellular homeostasis.

The response mechanism to heavy metals is highly complex, involving a multitude of genes. Comparative transcriptome analysis has shown that transcript abundance of at least 50% of genes differed between transgenic and wild-type plants, affecting the ROS-response system and various signaling pathways. According to KEGG enrichment analysis, more than 11 distinct categories were significantly upregulated in *CtDXS1*_*CtDXR1* transgenic plants, laying the groundwork for identifying molecular modules associated with heavy metal tolerance. Notably, multiple signaling pathways were enriched, including the plant hormone signal transduction, plant-pathogen interaction, phenylpropanoid biosynthesis pathway, suggesting potential regulatory crosstalk among various pathways. Further PPI analysis indicated AUX1 and CP1 might act as central regulators in Pb tolerance, offering candidate targets for future crop genetic engineering. Numerous proteins interacting with AUX1 and CP1, such as MYC, MPK, and Hsp, are involved in various signaling pathways during growth, development, and abiotic stress response, supporting the notion of significant crosstalk among different signaling pathways. For example, HSFA6b functions as a downstream regulator in the ABA-mediated heat stress response in *Arabidopsis* (Huang et al., 2016). Intriguingly, AUX1 and CP1 interacted with two phenylalanine ammonia-lyases (PALs), suggesting their potential role as key inducers of phenylpropanoid metabolism under Pb stress. Glutathione S-transferases (GSTs) are responsive to ABA and play roles in various aspects of abiotic stress, particularly in the detoxification process (Cao et al., 2021; Jiang et al., 2010; Wang et al., 2023b). The comparative transcriptome analysis also suggested that ABC transporters, ATPases, and metallothioneins might facilitate the translocation and uptake of Pb. ABC transporters are crucial for sequestering toxic metals in plants (Chang et al., 2022; Zhao et al., 2022). In *Arabidopsis*, functional nitrate transporters can reduce Pb uptake (Zhu et al., 2019). Heavy metal ATPases enhance heavy metal tolerance and phytoremediation by transporting heavy metals to intracellular locations or detoxifying them (Grønberg et al., 2021; Ma et al., 2021; Wang et al., 2007). Additionally, the transcript level of ATPase was increased in cucumber roots after ABA treatment (Janicka-Russak and Kabała, 2012). Metallothioneins play significant roles in Pb sequestration and confer heavy metal tolerance (de Francisco et al., 2023; Pirzadeh and Shahpiri, 2016). Current evidence also indicates that channels proteins (Moon et al., 2019; Wojas et al., 2007) are involved in the absorption and translocation of Pb. Compared with wild-type plants, the regulated expression of ABC transporters, ATPases, and metallothioneins in transgenic plants was consistent with the observed reduction in Pb uptake. However, further functional investigation of these components is warranted. Additionally, two SOD genes were upregulated in transgenic plants compared to wild-type plants. Together with transcriptomic data and enzymatic assays, the enhanced transcriptional levels and activities of the antioxidant system in transgenic plants collectively provided robust protection against Pb stress. Overall, the molecular networks underpinning the function of *CtDXS1* and *CtDXR1* in Pb defense are intricate, and the connections among various signaling pathways need further elucidation.

Beyond intrinsic plant metabolism and molecular adaptation, interactions between plants and their rhizosphere microbial communities also influence plant growth and heavy metal uptake, contributing to overall tolerance (Muehe et al., 2015). Plants can shape their surrounding microorganisms by secreting phytohormones such as ABA to foster a beneficial rhizosphere microbial community (Lopes et al., 2023), which in turn enhance plant growth and stress resilience (Tang et al., 2024). In our study, the diversity and structure of rhizosphere microorganisms associated with both transgenic and wild-type plants was investigated. *Proteobacteria*, for example, was identified as the most abundant group in the rhizospheres of various plants, including maize (Zhao et al., 2021), mangroves (Quintero et al., 2022), and Berchemia polyphylla (Tang et al., 2024). Cyanobacteria, recognized as their phototrophic, plant growth-promoting, and stress-tolerant functions, were also reported to be abundant (Wang et al., 2023a; Ullah et al., 2019). Furthermore, an increase of *Actinobacteriota* correlated with mitigation of heavy metal pollution in soils (Li et al., 2023, 2024). In this work, Pb stress led to a decrease in the diversity of the rhizosphere microbial community in both wild-type and transgenic plants, albeit to varying degrees (**Supplementary Table S5**). A similar decline in microbial diversity with increased heavy metal levels was also observed in *Tamarix ramosissima* rhizosphere (Qian et al., 2022). Intriguingly, transgenic plants with higher ABA levels exhibited a smaller reduction in rhizosphere microbial diversity compared to wild-type plants when grown in Pb-contaminated soil. Additionally, a variety of microorganisms associated with Pb tolerance were more prevalent in the rhizosphere of transgenic plants, potentially aiding root growth and reducing Pb uptake. For instance, transgenic plants maintained a relative stability in the diversity of *Proteobacteria* and *Actinobacteriota* following Pb exposure. Han et al. (2020a) noted that two strains from the phyla *Proteobacteria* reduced Pb accumulation in plants by facilitating Pb immobilization. Hence, the maintained abundance of *Proteobacteria* in the rhizosphere of transgenic plants may be linked to their enhanced tolerance to Pb (Zuo et al., 2023). Additionally, the increased abundance of heavy-metal-resistant genera, such as *Methylophilus*, *Massilia* and *Bradyrhizobium*, in transgenic plants likely contribute to the biodiversity in the Pb-polluted soil and may serve as biomarkers for the Pb-tolerant rhizosphere. Consistent with our hypothesis, as shown in **Figure 2**, the transgenic plants demonstrated greater biomass and lower Pb uptake compared to the wild-type plants. These findings suggest that limiting Pb uptake and enhancing antioxidant defenses are central to Pb tolerance observed in *CtDXS1_CtDXR1* transgenic plants. Additionally, we observed that the co-expression of these genes could confer drought tolerance, thereby broadening their potential applications in future crop improvement (data to be prepared separately).

Derived from the literature and our results, it is important to summarize the multifaceted roles of ABA in response to various heavy metals in plants. These roles include: (1) Modulating the production of endogenous plant hormones and signaling pathways (Guo et al., 2021; Ma et al., 2024; Zhang et al., 2025). (2) Influencing the expression of metal transporter genes, thereby facilitating transport heavy metals intracellularly or detoxify them (Kim et al., 2022; Janicka-Russak and Kabała, 2012; Zhao et al., 2023; Dhara and Raichaudhuri, 2021; Ma et al., 2021). (3) Regulating downstream signaling that directly or indirectly enhances antioxidant enzyme activities, contributing to cellular detoxification (Jalmi et al., 2018; Tian et al., 2023). (4) Remodeling the rhizosphere microorganisms to improve adaptation to abiotic stress (Tang et al., 2022).

**Figure 11:**
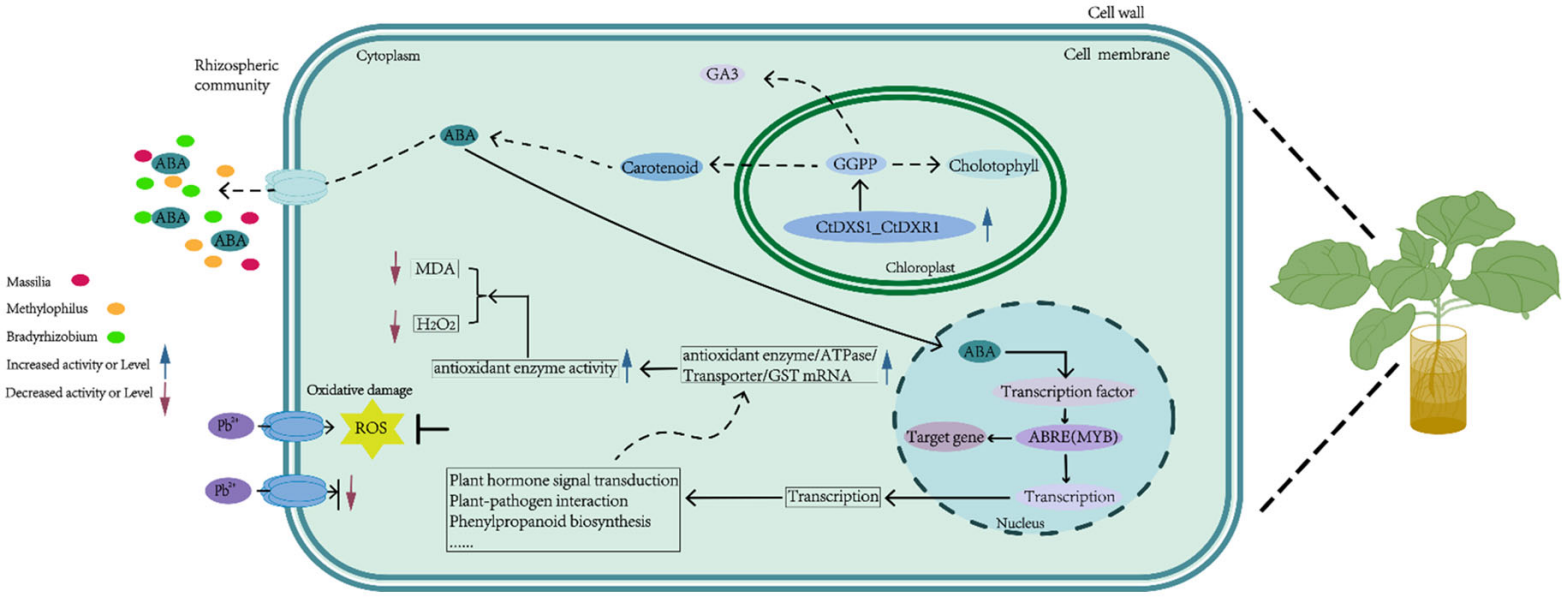
Proposed model for enhanced Pb tolerance in *CtDXS1*_*CtDXR1* transgenic plants.

Finally, we propose a potential molecular model to explain the enhanced Pb tolerance in *CtDXS1*_*CtDXR1* transgenic plants. In this framework, the co-expression of the *CtDXS1* and *CtDXR1* genes primarily boosts ABA accumulation. Subsequently, ABA remodels gene expression involved in various plant signaling pathways and metabolic processes, including plant hormone signal transduction, plant-pathogen interaction, phenylpropanoid biosynthesis etc. Within this network, AUX1 and CP1 may function as core regulators, operating individually and (or) cooperatively to induce downstream genes encoding transporter, ATPase and antioxidant enzymes. This effect contributes to the limitation of Pb uptake and improved activities of antioxidant enzymes. Concurrently, the ABA-mediated changes in rhizosphere microbial community further protect plants against Pb stress by reducing the uptake of Pb in plants and promoting plant growth (**Figure 11**).

## 5 Conclusion and prospective

To the best of our knowledge, this study provides the first evidence that genes in the MEP pathway, specifically CtDXS1 and CtDXR1, contribute to Pb tolerance in plants. This finding provides a promising approach for utilizing genetic manipulation of the MEP pathway and its secondary metabolites to address environmental Pb stress. We indicated that the enhanced Pb tolerance observed in transgenic plants are involved in three interconnected strategies, increase in antioxidant enzyme activities, decrease in Pb uptake and enrichment of Pb-tolerant microbes. AUX1 and CP1 might be core regulators of responsive signaling networks governing the adaption to Pb stress.

Future investigation should delve deeper into the phytochemistry, physiology, and molecular mechanisms underpinning this phenomenon. First, it is necessary to qualify the ABA content in the rhizospheric environment to provide critical insights into its role in plant-microbe interactions under Pb stress. Second, transgenic plants harboring the potential key regulators, such as *AUX1* and *CP1* genes, will be engineered, followed by an assessment of their Pb tolerance. Third, apart from the four kinds of isoprenoid products in this study, a metabolomic approach will be employed to explore the full spectrum of downstream metabolites derived from the MEP pathway, thereby elucidating the holistic function of *CtDXS1*_*CtDXR1* gene group in Pb tolerance. Finally, apart from model plant *N. benthamiana*, a wider range of crops harboring *CtDXS1* and *CtDXR1* genes will be utilized to assess their roles in Pb tolerance, and thereby the application of *CtDXS1* and *CtDXR1* genes might be enlarged.

## Data Availability

The original contributions presented in the study are included in the article/**Supplementary Material**. Further inquiries can be directed to the corresponding author/s.

## Supplementary material

Supplementary Figure 1 The schematic diagram of terpenoid, such as ABA, biosynthesis via MEP pathway. Enzyme abbreviations are as follows: DXS: 1-deoxy-D-xylulose-5-phosphate synthase, DXR: 1-deoxy-d-xylulose-5-phosphate reductoisomerase, MCT: MEP cytidyl-transferase, CMK: 4-(cytidine 5′-diphospho)-2C-methyl-d-erythritol kinase, MDS: 2C-methyl-d-erythritol-2,4- cyclodiphosphate synthase, HDS: 4-hydroxy-3-methylbut-2-enyl diphosphate synthase, HDR: 4-hydroxy-3-methylbut-2-enyl diphosphate reductase, IDI: isopentenyl diphosphate isomerase. Intermediate abbreviations are as follows: GAP: d-glyceraldehyde- 3-phosphate, DXP: 1-deoxy-d-xylulose 5-phosphate, MEP: 2-*C*-methyl-D-erythritol-4-phosphate, CDP-ME: 4-diphosphocytidyl-2-C-methyl-D-erythritol, CDP-MEP: 4-diphosphocytidyl-2-C-methyl-D-erythritol 2-phosphate; MEcDP: 2-C-methyl-d-erythritol-2,4-cyclodiphosphate, HMBDP: 1-hydroxy-2-methyl-2-(E)-butenyl-4-diphosphate, IDP: isopentenyl diphosphate, DMADP: dimethylallyl diphosphate.

Supplementary Figure 2 The schematic diagram of dual-transgene vector construction. The modified pCambia1301 vector contained two separate expression cassettes (A and B). Only commonly used *Bgl*II, *BstE*II, and *Kpn*I recognition sites are shown. For cassette A, the *Bgl*II site is located at the downstream of 35S promoter and the *BstE*II site is located at the upstream of NOS terminator, whereas cassette B contains an internal *Kpn*I site. During the construction of dual-transgene vector, *CtDXR1* gene was firstly inserted into cassette A using double restriction enzyme (*Bgl*II/*BstE*II). Then, the *CtDXS1* gene was inserted into the cassette B using homology recombination based on *Kpn*I site.

Supplementary Figure 3 Expression profiles of *CtDXS1* and *CtDXR1* genes under Pb exposure for 7 days. (A) Real-time PCR result of *CtDXS1* gene; (B) Real-time PCR result of *CtDXR1* gene.

Supplementary Figure 4 The PCR verification results of wild-type and *CtDXS1*_*CtDXR1* transgenic plants using DNA as templates. (A) The PCR verification of *CtDXS1* gene in pCambia1301: *CtDXR1_CtDXS1* plasmid. Lane 1, 2, and 3 represented PCR products of pCambia1301: *CtDXR1_CtDXS1* plasmid, respectively, while lane 4 represented PCR product of water; (B) The PCR verification of *CtDXS2* gene in pCambia1301: *CtDXR1_CtDXS1* plasmid. Lane 1, 2, and 3 represented PCR products of pCambia1301: *CtDXR1_CtDXS1* plasmid, respectively, while lane 4 represented PCR product of water; (C) The PCR verification of *CtDXS1* gene in transgenic and wild-type plants. Lane 1, 2, and 3 represented PCR products of OE2, OE10 and wild-type plants, respectively; (D) The PCR verification of *CtDXR1* gene in transgenic and wild-type plants. Lane 1, 2 and 3 represented PCR products of OE2, OE10 and wild-type plants, respectively.

Supplementary Figure 5 Relative expression level of *CtDXS1* and *CtDXR1* genes in OE2 and OE10 transgenic lines, respectively. All data are mean ± SD for no less than three biological replicates. ** and *** represent *P*<0.01 and *P*<0.001, respectively.

Supplementary Figure 6 The KEGG network analysis of the up-regulated DEGs in *CtDXS1*_*CtDXR1* transgenic plants under Pb stress.

Supplementary Figure 7 The relative abundance of rhizosphere microbial community at phyla level (A) and genus level (B), respectively. WTCK, WTPb, OECK, and OEPb represent wild-type plants under normal conditions, wild-type plants under Pb stress, transgenic plants under normal conditions, and transgenic plant under Pb stress, respectively.

Supplementary Table 1 The primers used in this study.

Supplementary Table 2 The statistical information of RNA libraries.

Supplementary Table 3 The significantly upregulated KEGG pathways in *CtDXS1*_*CtDXR1* transgenic *N. benthamiana*.

Supplementary Table 4 The predicted interaction network of the significant members involved in Pb tolerance.

Supplementary Table 5 Summary of the sequencing data and the alpha indices of prokaryotic communities.
