# Correction: Co-expression of *Cassia tora* 1-deoxy-D-xylulose-5-phosphate synthase and 1-deoxy-D-xylulose-5-phosphate reductoisomerase enhances tolerance of transgenic *Nicotiana benthamiana* to lead (Pb) stress

**DOI:** 10.3389/fpls.2025.1739234

**Published:** 2025-12-05

**Authors:** Xue Huang, Chunyao Tian, Zichun Ma, Jieru Chen, Hongting Liu, Wei Zhang, Zhongda Li, Pingping Lu, Leyao Wang, Hai Liao, Jiayu Zhou

**Affiliations:** 1 School of Life Science and Engineering, Southwest Jiaotong University, Chengdu, Sichuan, China; 2 Bureau of Longnan Business Environment Construction, Longnan, Gansu, China

**Keywords:** ABA, Cassia tora, Pb stress, MEP pathway, Nicotiana benthamiana

There was a mistake **Figure 11** as published. The corrected Figure 11 appears below. The original version of this article has been updated.

**Figure 11:**
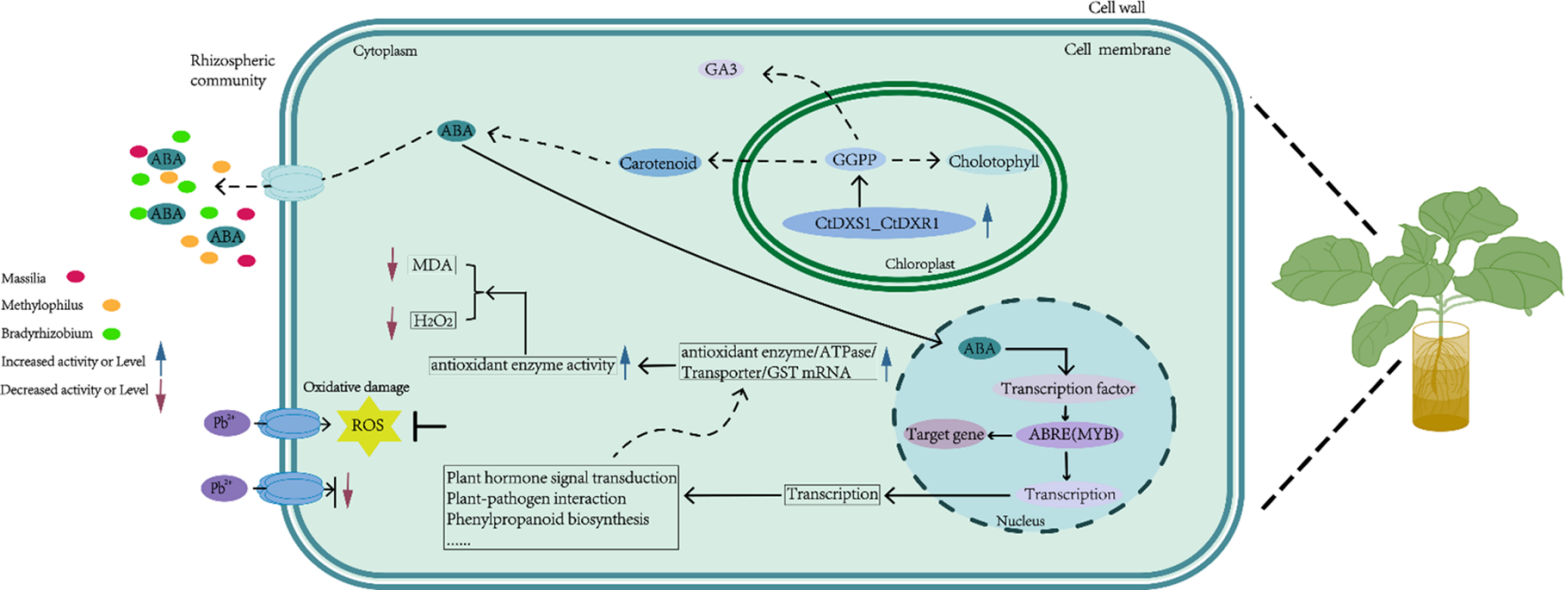
Proposed model for enhanced Pb tolerance in *CtDXS1_CtDXR1* transgenic plants.

