# Supplementary figures and images for: Co-expression of Cassia tora 1-deoxy-D-xylulose-5-phosphate synthase and 1-deoxy-D-xylulose-5-phosphate reductoisomerase enhances tolerance of transgenic Nicotiana benthamiana to lead (Pb) stress
Source: Front Plant Sci. 2025 Nov 4;16:1657368. doi: 10.3389/fpls.2025.1657368 (PMC12623350; doi:10.3389/fpls.2025.1657368)

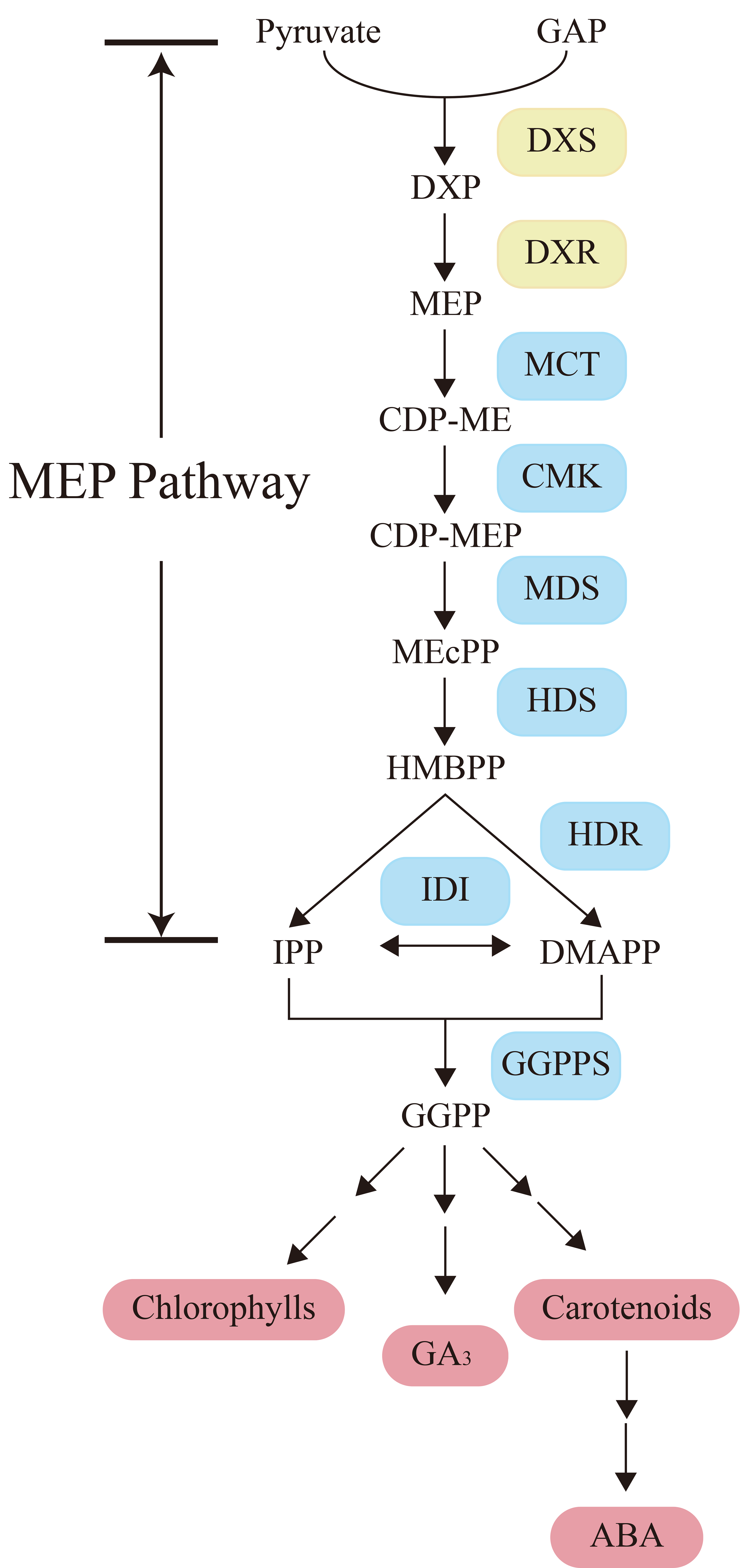

Supplement: Supplementary Figure 1 — The schematic diagram of terpenoid, such as ABA, biosynthesis via MEP pathway. Enzyme abbreviations are as follows: DXS: 1-deoxy-D-xylulose-5-phosphate synthase, DXR: 1-deoxy-d-xylulose-5-phosphate reductoisomerase, MCT: MEP cytidyl-transferase, CMK: 4-(cytidine 5′-diphospho)-2C-methyl-d-erythritol kinase, MDS: 2C-methyl-d-erythritol-2,4- cyclodiphosphate synthase, HDS: 4-hydroxy-3-methylbut-2-enyl diphosphate synthase, HDR: 4-hydroxy-3-methylbut-2-enyl diphosphate reductase, IDI: isopentenyl diphosphate isomerase. Intermediate abbreviations are as follows: GAP: d-glyceraldehyde- 3-phosphate, DXP: 1-deoxy-d-xylulose 5-phosphate, MEP: 2-C-methyl-D-erythritol-4-phosphate, CDP-ME: 4-diphosphocytidyl-2-C-methyl-D-erythritol, CDP-MEP: 4-diphosphocytidyl-2-C-methyl-D-erythritol 2-phosphate; MEcDP: 2-C-methyl-d-erythritol-2,4-cyclodiphosphate, HMBDP: 1-hydroxy-2-methyl-2-(E)-butenyl-4-diphosphate, IDP: isopentenyl diphosphate, DMADP: dimethylallyl diphosphate. [file Image1.tif]

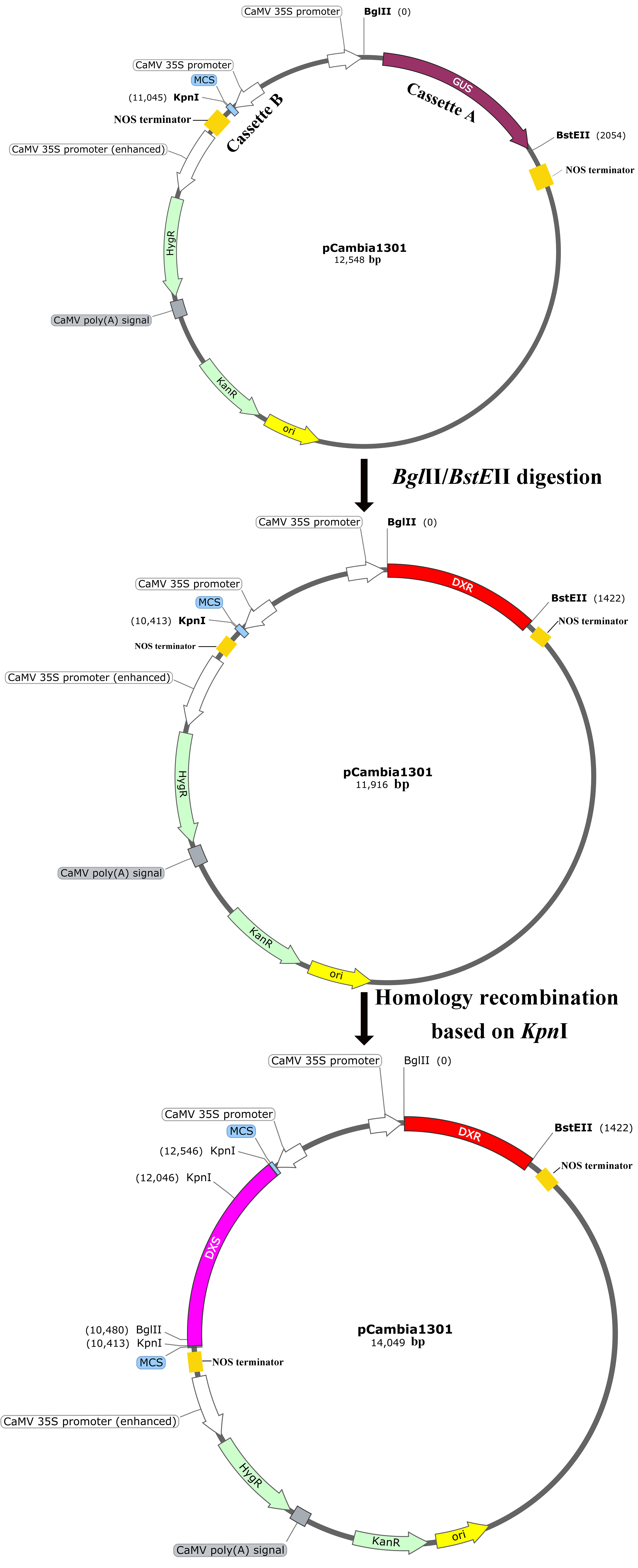

Supplement: Supplementary Figure 2 — The schematic diagram of dual-transgene vector construction. The modified pCambia1301 vector contained two separate expression cassettes (A and B). Only commonly used BglII, BstEII, and KpnI recognition sites are shown. For cassette A, the BglII site is located at the downstream of 35S promoter and the BstEII site is located at the upstream of NOS terminator, whereas cassette B contains an internal KpnI site. During the construction of dual-transgene vector, CtDXR1 gene was firstly inserted into cassette A using double restriction enzyme (BglII/BstEII). Then, the CtDXS1 gene was inserted into the cassette B using homology recombination based on KpnI site. [file Image2.tif]

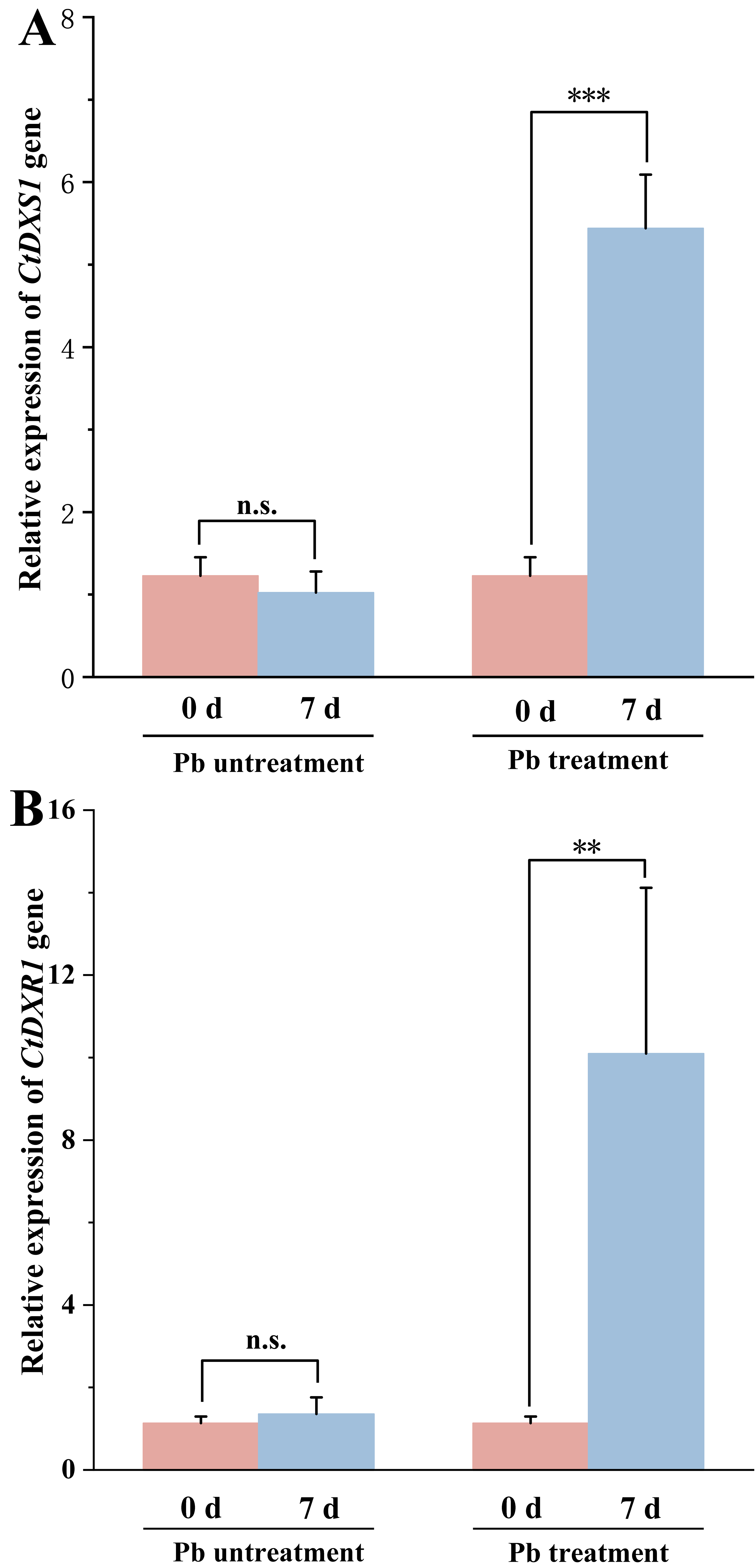

Supplement: Supplementary Figure 3 — Expression profiles of CtDXS1 and CtDXR1 genes under Pb exposure for 7 days. (A) Real-time PCR result of CtDXS1 gene; (B) Real-time PCR result of CtDXR1 gene. [file Image3.tif]

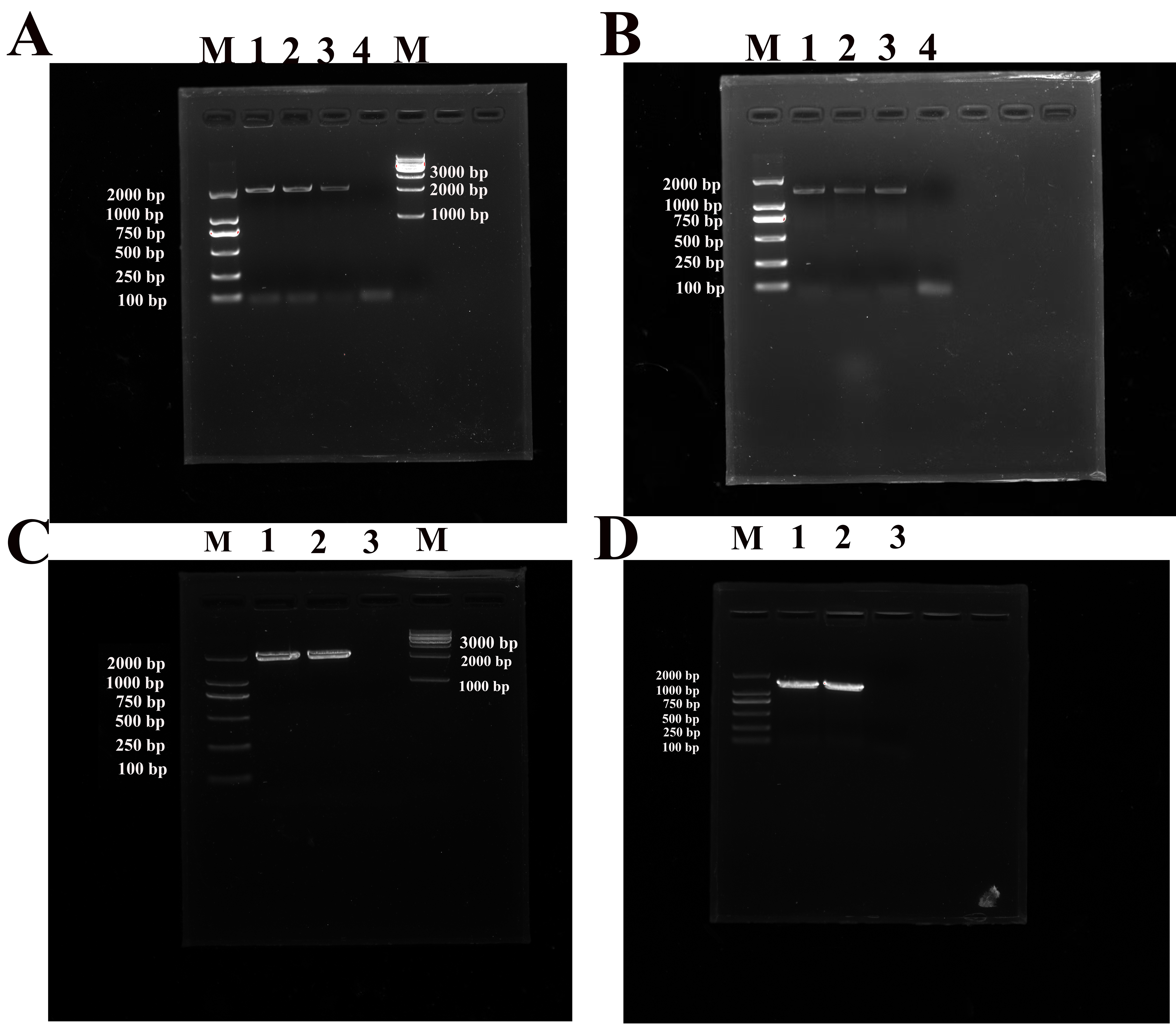

Supplement: Supplementary Figure 4 — The PCR verification results of wild-type and CtDXS1_CtDXR1 transgenic plants using DNA as templates. (A) The PCR verification of CtDXS1 gene in pCambia1301: CtDXR1_CtDXS1 plasmid. Lane 1, 2, and 3 represented PCR products of pCambia1301: CtDXR1_CtDXS1 plasmid, respectively, while lane 4 represented PCR product of water; (B) The PCR verification of CtDXS2 gene in pCambia1301: CtDXR1_CtDXS1 plasmid. Lane 1, 2, and 3 represented PCR products of pCambia1301: CtDXR1_CtDXS1 plasmid, respectively, while lane 4 represented PCR product of water; (C) The PCR verification of CtDXS1 gene in transgenic and wild-type plants. Lane 1, 2, and 3 represented PCR products of OE2, OE10 and wild-type plants, respectively; (D) The PCR verification of CtDXR1 gene in transgenic and wild-type plants. Lane 1, 2 and 3 represented PCR products of OE2, OE10 and wild-type plants, respectively. [file Image4.tif]

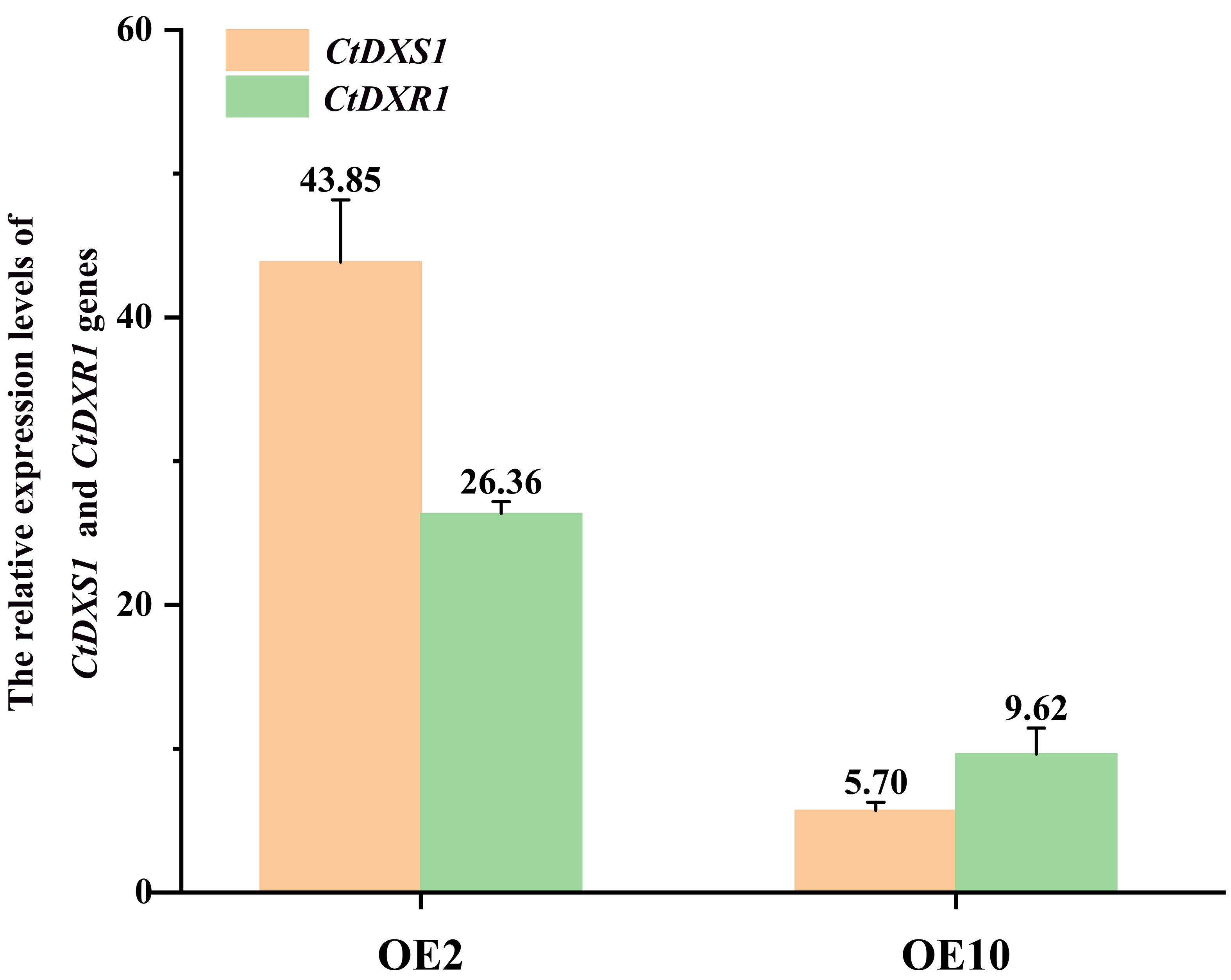

Supplement: Supplementary Figure 5 — Relative expression level of CtDXS1 and CtDXR1 genes in OE2 and OE10 transgenic lines, respectively. All data are mean ± SD for no less than three biological replicates. ** and *** represent P<0.01 and P<0.001, respectively. [file Image5.tif]

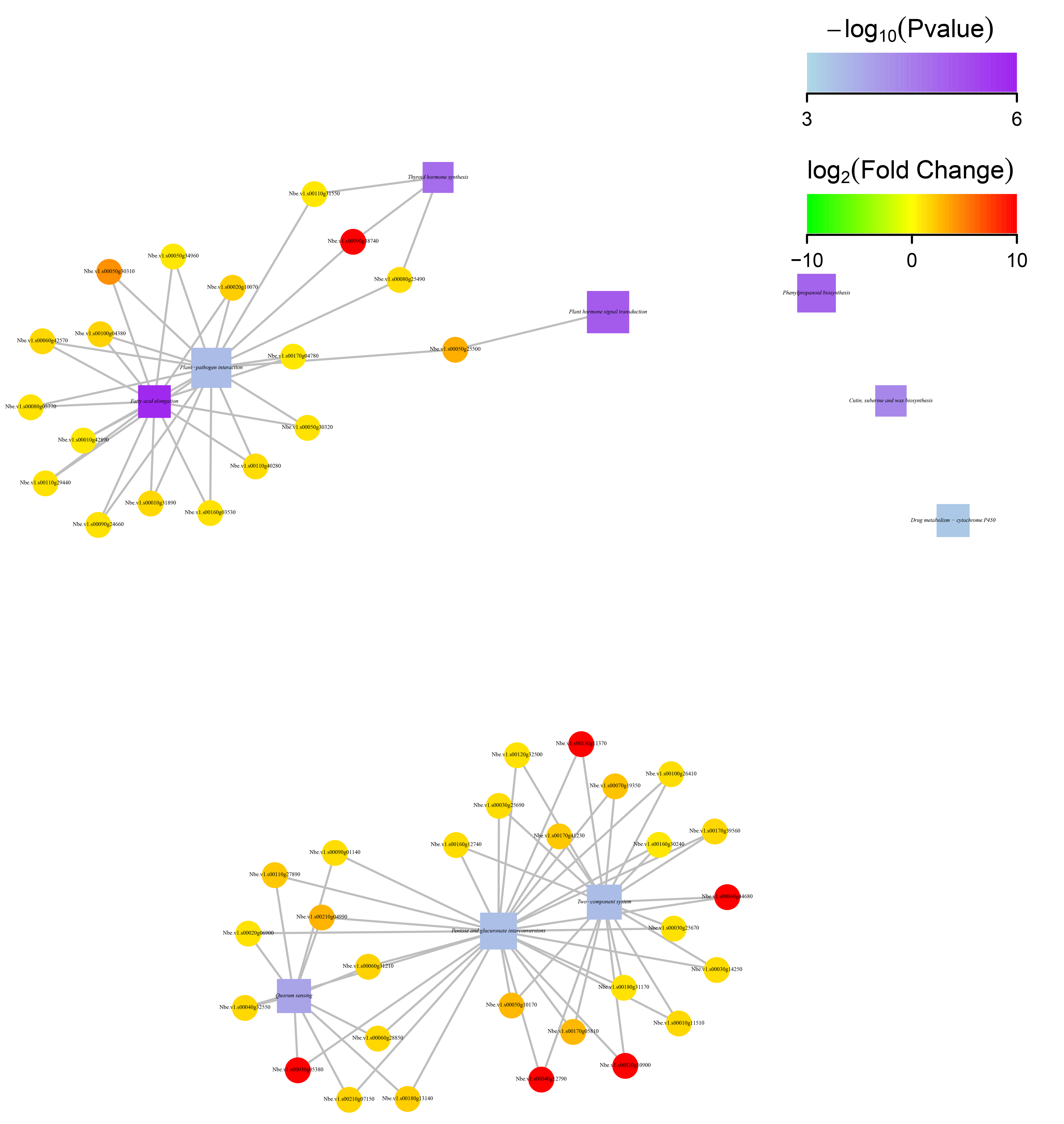

Supplement: Supplementary Figure 6 — The KEGG network analysis of the up-regulated DEGs in CtDXS1_CtDXR1 transgenic plants under Pb stress. [file Image6.tif]

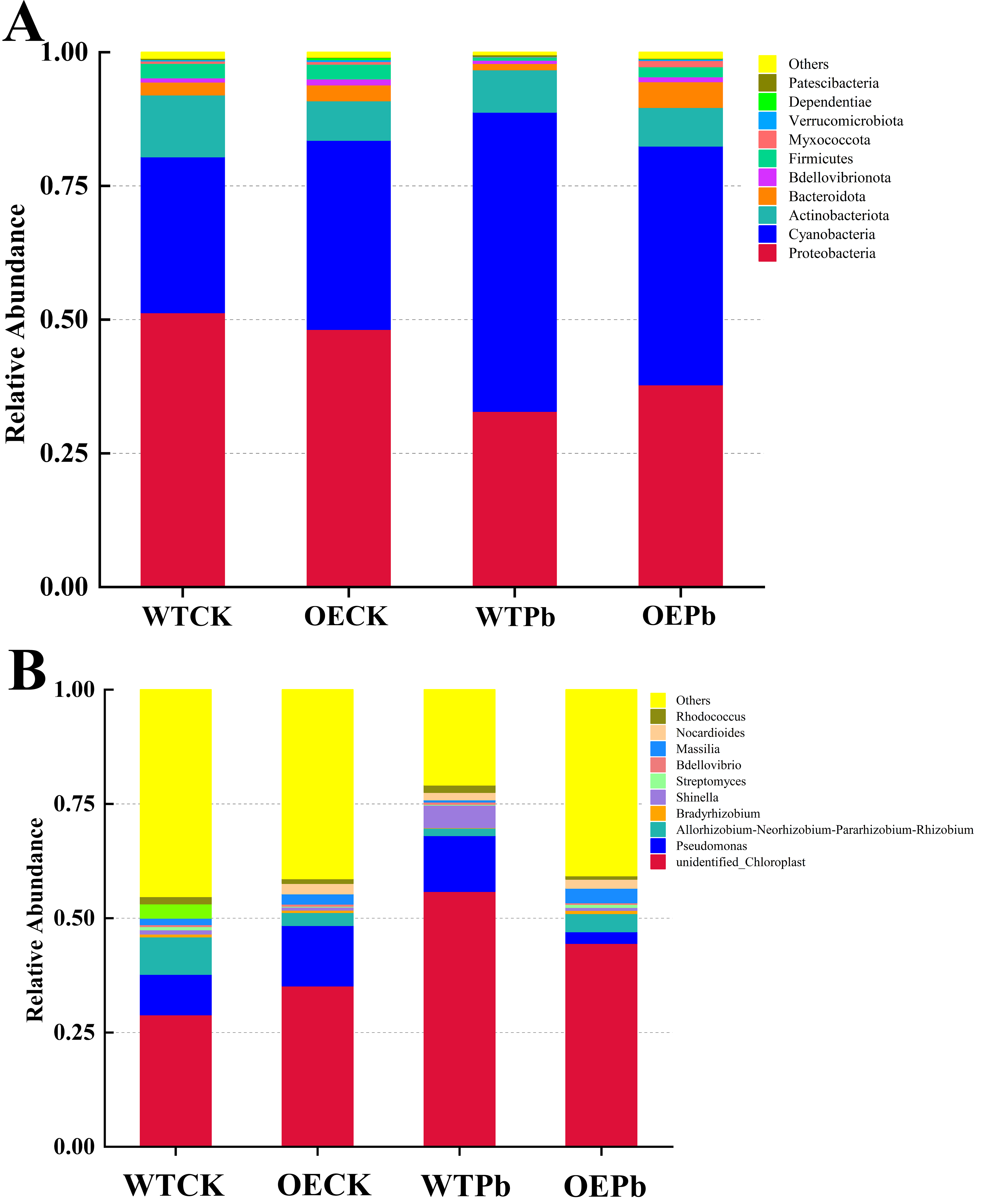

Supplement: Supplementary Figure 7 — The relative abundance of rhizosphere microbial community at phyla level (A) and genus level (B), respectively. WTCK, WTPb, OECK, and OEPb represent wild-type plants under normal conditions, wild-type plants under Pb stress, transgenic plants under normal conditions, and transgenic plant under Pb stress, respectively. [file Image7.tif]
